# Delivering the future of immunotherapy: A state-of-the-art review of gene editing in immune cells with lipid nanoparticles

**DOI:** 10.1016/j.mtbio.2026.103334

**Published:** 2026-06-08

**Authors:** Ivan Ciganek, Christophe Delehedde, Anne Galy, Michel Cogné, Nathalie Rameix, Catalina Bordeianu, Chantal Pichon

**Affiliations:** a Sanofi R&D, Vitry-sur-Seine, 94400, France; b INTHERNA/ART ARNm, Inserm, Orleans, 45000, France; c University of Orléans, Orleans, 45000, France; d Institut Universitaire de France, Paris, 75000, France; e ART-TG, Inserm, Evry, France; f MOBIDIC, Inserm, Rennes, France

## Abstract

CRISPR-based genome editing has rapidly advanced immune cell engineering, creating opportunities to enhance functionality, persistence, and specificity in cancer, infectious, and autoimmune diseases. The translation of these strategies depends critically on safe and efficient delivery systems. Viral vectors achieve high efficiency, but face limitations related to immunogenicity, genomic integration, and manufacturing scalability. Non-viral methods such as electroporation are effective for *ex vivo* editing yet remain unsuitable for *in vivo* use. Lipid nanoparticles (LNPs) have emerged as a clinically validated, scalable, and biocompatible alternative capable of delivering CRISPR cargo in the form of DNA, mRNA, or ribonucleoproteins. Recent innovations, including selective organ targeting, ligand conjugation, and sequence-level translational control are expanding their precision and versatility in immune cell engineering. Despite these advances, major challenges remain, including suboptimal endosomal escape, extrahepatic targeting, and immune trade-offs such as the PEG dilemma. By critically synthesizing current progress and limitations, this review highlights the central role of LNPs in enabling CRISPR-based immune engineering and outlines future directions for clinical translation**.**

## Graphical abstract

**Figure:**
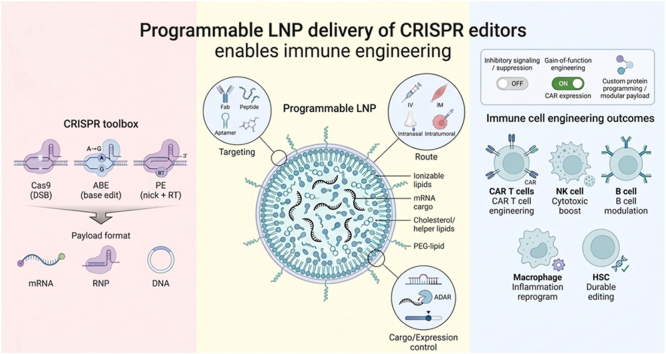

## 1 Introduction

Gene editing has emerged as a transformative approach in biotechnology and medicine, enabling precise genome modification with applications spanning from disease modeling to therapeutic interventions [1]. Among the numerous applications of gene editing, immune cell engineering holds particular promise for enhancing cell functionality, persistence, and specificity [2]. This advancement is particularly impactful in cancer immunotherapy, autoimmune diseases, and infectious diseases, with therapies like chimeric antigen receptor (CAR)-T cells demonstrating groundbreaking clinical outcomes [3,4].

At the core of these advancements are the Clustered regularly interspaced short palindromic repeats (CRISPR)-CRISPR-associated protein (Cas) system and its variants, which have surpassed previous gene-editing technologies, revolutionizing gene editing with their unparalleled specificity, efficiency, and convenience [5,6]. These systems, particularly CRISPR-Cas9, have been widely adopted for genetic modifications and corrections in genetic disorders, culminating in Casgevy, the first ever CRISPR-based gene therapy to receive regulatory approval [7]. Beyond hematopoietic disorders, CRISPR is now transforming immune cell engineering, where T cells, B cells, Natural Killer (NK) cells, macrophages, dendritic cells (DCs) and hematopoietic stem cells (HSCs) can be precisely reprogrammed for improving immunotherapy outcomes.

However, the efficiency of CRISPR-based genome editing hinges on the utilization of appropriate delivery system to introduce gene-editing machinery into the targeted cells without compromising the cell viability and functions. Therefore, potential success of CRISPR-based genome editing depends on both the precision of the editing tools and the reliability of the delivery mechanisms [4,8]. While lentiviral (LV) and adeno-associated viral (AAV) vectors have achieved high delivery efficiency and durable gene expression, contributing to positive clinical outcomes, their use is still limited by concerns over immunogenicity, insertional mutagenesis, and scalability in manufacturing [9]. Moreover, the need for transient, on-target and potentially re-dosable genome editing, particularly in immune cells, has intensified interest in non-viral strategies that maintain efficiency, while offering greater safety and regulatory flexibility [10,11]. Most common non-viral physical approach is electroporation (EP), limited only to *ex vivo* applications [12].

Lipid nanoparticles (LNPs) have emerged as the leading non-viral nanoparticle-(NP) based platform for delivering nucleic acids, including CRISPR components, owing to their high payload capacity, ease of formulation, and clinical scalability. Their wide success and application in billions of people in mRNA-based COVID-19 vaccines has further highlighted their biocompatibility and potential in gene (immuno)therapy [13,14]. However, despite these advantages, LNP-mediated gene editing still faces critical challenges, such as suboptimal endosomal escape efficiency and the need for precise targeting strategies [15].

This comprehensive review examines the current state and future directions in genome editing technologies and (LNP) delivery systems that enable their application in immune cells, driving new strategies in next-generation immunotherapy. Furthermore, we critically assess the current challenges related to LNP technology, explore emerging solutions such as multifunctional and AI-designed LNPs, and discuss the future of gene editing in clinical applications. By synthesizing insights from recent high-impact studies, this paper aims to provide a comprehensive and analytical perspective on the evolving landscape of LNP-mediated gene editing of immune cells.

## 2 Mechanism and modalities of CRISPR-Cas gene editing

CRISPR-Cas system was originally discovered as a type of adaptive defense mechanism in prokaryotes against external infections. It has since been repurposed as a powerful RNA-guided platform for genome editing and modulation, ultimately yielding Jennifer Doudna and Emmanuelle Charpentier the 2020 Nobel Prize in Chemistry [[16], [17], [18]]. The system relies on ribonucleoprotein (RNP) complex made of Cas9 protein and a synthetic single-guide RNA (sgRNA), which combines CRISPR-RNA (crRNA) for target specificity; and *trans*-activating CRISPR RNA (tracrRNA) for RNP assembly and activation. Target recognition is initiated through complementary base pairing between the sgRNA and a 20-nucleotide protospacer within the DNA, a protospacer adjacent motif (PAM), a sequence flanking the target site that is specific to the Cas enzyme. PAM binding triggers local DNA melting, allowing sgRNA-DNA hybridization and activating Cas9 nuclease domains - HNH and RuvC, which induce a double-strand break (DSB). Conventionally, these breaks are repaired through either non-homologous end joining (NHEJ), which introduces small insertions or deletions at the cleavage site (resulting in the knockout (KO) if the target is within protein coding region); or homology-directed repair (HDR), which enables genetic modifications using a donor (exogenous) DNA template [19].

Soon after elucidating the mechanism by which CRISPR-Cas9 works, the technology was applied for gene editing in eukaryotic cells and organisms, positioning CRISPR as the third and most transformative generation of gene editing tools, eclipsing earlier platforms like zinc-finger nucleases (ZFNs) and transcription activator-like effector nucleases (TALENs) [[20], [21], [22]]. Both ZFNs and TALENs use sequence-specific DNA-binding proteins to recognize the targeted DNA site followed by cutting, resulting in laborious customization of DNA-binding domains for each target [23]. Conversely, CRISPR technology offers more straightforward and adaptable approach as it utilizes short, easily adjustable sgRNA, complementary to targeted DNA region. The ease of programmability, combined with high cutting efficiency, positioned CRISPR-Cas9 at the forefront of genome engineering, with multiple attempts to use the technology for disease treatment, within just a few years of its debut [6,24]. Early proof-of-concept studies rapidly harnessed CRISPR for gene editing in immune cells, particularly T cells, demonstrating its therapeutic potential [[25], [26], [27]]. This progress culminated in initiation of clinical trials employing CRISPR-Cas for immunotherapy within just a few years since its first application. In more detail, after showing efficient disruption of checkpoint inhibitor programmed death-1 (PD-1) receptor in T cells, this strategy was soon applied in first-ever CRISPR clinical trial, initiated in 2016, in which patients with refractory non-small-cell lung cancer received CRISPR-Cas9 PD-1-edited T cells, showcasing safety and feasibility to use gene editing approaches [28]. Shortly after, additional clinical trials and applications, supported by increasing number of relevant research, expanded to immune and progenitor cells for cancer and human immunodeficiency virus type 1 (HIV-1) infection treatments, marking the beginning of a rapid expansion in gene editing-based immunotherapies and further solidifying the convergence of these two fields in clinical development [29,30].

### 2.1 The CRISPR toolbox

#### 2.1.1 Cas9

To date, several representative members from each subtype of Cas-based CRISPR systems have been implemented for genome engineering in eukaryotes. Still, CRISPR-Cas9 represents the benchmark in gene editing, and the two most widely used Cas9 proteins are ones derived from *Streptococcus pyogenes* (SpCas9) and *Staphylococcus aureus* (SaCas9). These orthologs recognize distinct PAM sequences: SpCas9 recognizes NGG PAM sequence immediately next to the 3'end of targeted DNA, whereas SaCas9 recognizes a NNGRRT PAM [where R represents a purine (i.e., A or G)]. Beyond its role in target cutting specificity, the PAM defines the regions accessible to a given Cas protein, with SpCas9's widely occurring NGG PAM offering greater target flexibility, compared to the more restrictive PAM of SaCas9. Another important difference is in size: SaCas9 (139 kDa with coding sequence of 3.2 kb) *versus* SpCas9 (158 kDa and coding sequence of 4.1 kb), which can be an important factor for applications where packaging constraints limit the choice of gene-editing tools [19,31]. On-target specificity is another major factor to consider, as initial CRISPR-Cas9 applications raised concerns with off-target cleavage resulting in unintended mutations [32]. In response, improved CRISPR variants were synthesized with enhanced specificity and safety, such as high-fidelity Cas9 nucleases with point mutations (e.g., SpCas9-HF1) that showed reduced off-target cuts while retaining on-target activity [33].

**Fig. 1:**
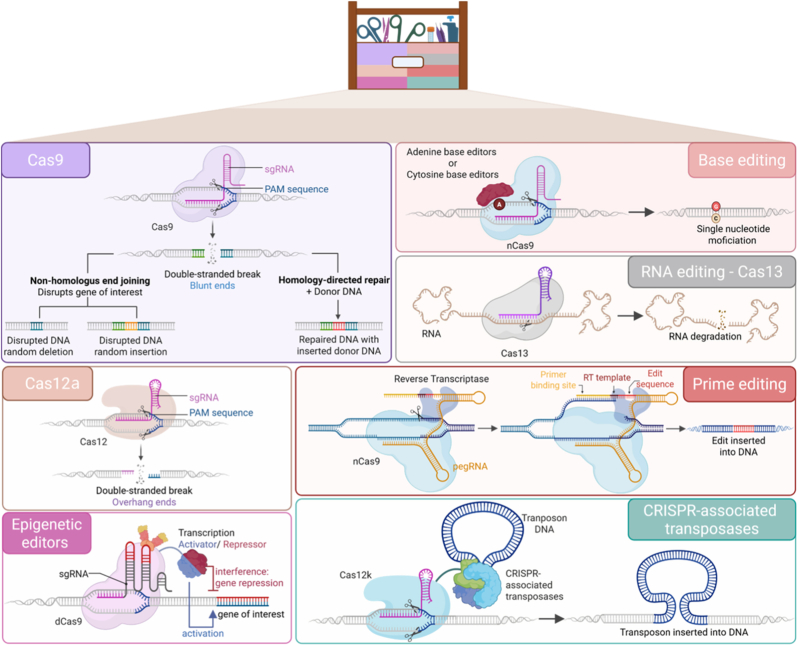
The CRISPR toolbox for genome engineering.

At the same time, different types and variants of CRISPR-Cas systems (Fig. 1), with different origins, as well as newly engineered constructs have been employed to expand gene-editing capabilities and address existing shortcomings, providing a versatile toolbox for diverse applications, especially in immunotherapy.

#### 2.1.2 Cas12a

A significant addition to CRISPR toolbox was Cas12a (Cpf1), a Class II CRISPR nuclease with a T-rich PAM requirement, as opposed to the Cas9 enzymes which require purine rich-PAM. This distinction broadens the range of genomic sites accessible for editing. Notably, Cas12a displays a staggered-cut DNA cleavage mechanism resulting in ’sticky’, overhang ends, which is distinct from blunt ends induced by SpCas9. This feature can enhance the efficiency of gene insertion through HDR [34]. Cas12a's ability to autonomously process multiple gRNAs from a single transcript allows efficient multiplex editing - an asset for immune cells, where simultaneous knock out (KO) of several genes [e.g. T cell receptor (TCR), major histocompatibility complex (MHC), inhibitory receptors in T cells] can improve the efficacy of cell-based therapies [35].

#### 2.1.3 Cas13

Beyond DNA editing, RNA-targeting CRISPR systems, particularly Cas13 enzymes, have opened entirely new avenues for RNA editing, selective RNA degradation or transcript modulation. The type IV CRISPR-Cas13 system specifically binds and cleaves single-stranded RNA, enabling transcript knockdown without altering genomic DNA, reducing concerns over permanent off-target mutations [35]. Cas13d has been employed in T cells through the multiplexed effector guide arrays (MEGA) platform to achieve precise and reversible transcriptomic regulation. This approach enables targeted KO of inhibitory receptors and metabolic regulators, thereby reducing T cell exhaustion and enhancing CAR T cell persistence and functionality​ [36].

#### 2.1.4 Base editors (BE)

CRISPR systems have been further refined to enable precise genetic modifications, even at the resolution of single nucleotide, without having to rely on inducing DSB and cell's repair machinery. Base editing enables the direct conversion of one DNA base into another by employing Cas9 nickase (nCas9), which can nick one DNA strand, fused to a deaminase enzyme to achieve single nucleotide polymorphisms (SNPs). sgRNA-nCas9 complex guides either cytosine base editors (CBEs) or adenine base editors (ABEs) that enable efficient C:G to T:A and A:T to G:C transitions, respectively [37]. By installing premature stop codons or disrupting splice sites, BEs can effectively knock out genes or modulate gene expression with greater predictability and efficiency than traditional Cas9-induced indels. This strategy is extensively explored in the context of T cell engineering by targeting multiple genes (such as *PD-1* and *T cell receptor alpha constant*, *TRAC*) relevant for production of CAR T cell therapies. Growing amount of research showcased efficient multiplex editing in T cells and NK cells, achieving KOs with fewer chromosomal translocations, than conventional Cas9 editing, and eventually resulting in enhanced T & NK cell functionality [[38], [39], [40], [41]]. Notably, a recent first-in-human use of base-edited CAR T cells demonstrated promising remission in a patient with T cell leukemia, highlighting the clinical potential of this approach to generate allogeneic, “off-the-shelf” cell therapies [42]. Another testament to the field's rapid evolution is the development of photoactivatable RNA base editors such as PA-rABE, which leverage Cas13 enzymes and light-inducible deaminases to enable precise, reversible RNA editing. This system has demonstrated efficient, low-off-target transcript modulation both *in vitro* and *in vivo* [43].

#### 2.1.5 Prime editors (PE)

As an extension of precision editing tools, prime editing was developed to further expand the versatility of CRISPR-based systems by enabling a wide variety of targeted edits, including all possible base-to-base conversions, small insertions, and deletions, without requiring DSBs or donor templates. This is achieved by fusing nCas9 to a reverse transcriptase (RT), guided by a specialized prime editing guide RNA (pegRNA) that both specifies the genomic target and encodes the desired edit. Upon binding, nCas9 introduces a nick in one DNA strand, allowing the RT to copy the edit directly into the genome in a template-dependent manner. In contrast to BE, PE are not limited to specific transition mutations and can achieve a broader range of edits (introduction of multiple premature STOP codons, splice site disruption, frameshift mutations, or deletion of large segments of regulatory or coding sequence) with high fidelity and reduced risk of undesired indels or chromosomal instability [44,45]. While still emerging, this approach has already demonstrated efficient editing in human T cells, including disruption of *TRAC* and Beta-2 microglobulin (B2M; commonly knocked out to prevent MHC class I expression), thus holding considerable promise for refining allogeneic CAR T cell therapies [46]. Ongoing improvements in pegRNA design and PE architecture are expected to enhance editing efficiency and expand the range of editable sequences, including the length of insertions. A notable demonstration of this is prime-editing-assisted site-specific integrase gene editing (PASSIGE), which includes prime editing coupled with integrases to achieve efficient, unidirectional, and template-free insertion of large DNA fragments at defined genomic loci. This approach offers a powerful and programmable platform for precise genome engineering, such as integration of a 3.5 kb CAR transgene cassette, with significant therapeutic potential [47]. Recent progress has introduced new systems called DNA polymerase editors (DPEs) and click editors (CEs), which differ from prime editing by using DNA polymerases and DNA templates instead of RT and RNA. These DNA polymerases are more precise and processive, and the DNA templates are more stable and easier to modify, resulting in more reliable editing and successful insertion of longer sequences [48]. Overall, prime editing continues to stand out as a promising tool for next-generation immunotherapies enabling accurate, flexible, and scarless genetic modifications.

#### 2.1.6 Epigenetic editors (EE)

Beyond direct sequence modification, CRISPR systems have been adapted to modulate gene expression by rewriting the epigenetic landscape without altering the underlying DNA. This is achieved by employing catalytically dead Cas9 (dCas9), fused to effector domains such as transcriptional repressors, activators, or chromatin-modifying enzymes. Guided by sgRNAs, these programmable epigenetic editors can target promoters, enhancers, or other regulatory elements to install or remove specific epigenetic marks, including DNA methylation (via DNMT3A/TET1) and histone modifications (via p300, LSD1, or KRAB) to either activate or silence gene expression. This approach is termed CRISPR interference (CRISPRi) or CRISPR activation (CRISPRa), respectively, and it allows us to switch genes off or on. Compared to permanent genetic KOs, epigenetic editing offers a potentially safer alternative, as it enables dynamic and tunable control of gene networks with minimal risk of DNA instability and breaks. Still, potential limitations of epigenetic editing include the inability to induce robust or sustained gene expression changes at certain loci, as well as the potential risks of off-target effects at distal genomic regions due to 3D chromatin architecture [49,50]. In immune cells, EEs have been used to reprogram exhaustion pathways, modulate cytokine profiles, and promote memory-like T cell states by regulating transcription without introducing genomic breaks. In detail, suppression of the H3K9 methyltransferase SUV39H1 improved long-term CAR T cell persistence protection against tumor rechallenges, highlighting the perspective of targeted epigenetic reprogramming in immunotherapy [51].

#### 2.1.7 Beyond “classical” CRISPR-based editing: compact enzymes, transposases, CRISPR-associated transposases, proprietary technology & bridge RNA

An emerging frontier beyond the classical CRISPR toolbox is the development of smaller Cas enzymes and proprietary nucleases designed to enhance delivery, specificity, and efficiency. Notable examples include CasΦ (Cas12j), Cas14, CasX (Cas12e), and CasY (Cas12d), all significantly smaller than Cas9 or Cas12a (100 – 150 kDa) [52,53]. For instance, CasΦ is only 70 - 80 kDa (half Cas9's size) yet retains robust DNA-cutting activity with an expanded range of target sequences due to the minimal PAM [54]. These naturally compact systems often require some engineering to maximize activity in mammalian settings. For example, wild-type Cas12f is barely active in human cells, but Xu et al. created CasMINI by protein and guide RNA engineering [55]. CasMINI (529 aa; ∼50 kDa) achieved genome editing and even base editing in human cells, with efficiencies comparable to Cas12a. The main advantage of these compact editors is their delivery compatibility, which can be extremely advantageous when combining Cas proteins with base, prime or epigenetic editors.

Unlike the precise gene editing tools discussed earlier, transposon systems such as Sleeping Beauty (SB) and piggyBac (pB), mediate gene addition through non-homologous integration, offering an alternative to HDR-dependent strategies. These are cut-and-paste DNA transposons that integrate transgenes into the genome via a transposase (Tns) enzyme, which excises the transposon from a donor plasmid and inserts it at TA- (SB) or TTAA-specific sites (pB), enabling stable integration of large genetic payloads (>10 kb). Unlike Cas9, which induces DSBs and depends on cellular repair, transposons directly integrate their cargo [56]. For example, the SB system has been used clinically to generate CD19 CAR-T cells, achieving up to 80% integration efficiency and long-term remission in leukemia patients [57]. Similarly, SB transposons have been applied to allogeneic CAR-T manufacturing in combination with CRISPR to produce “universal” CAR-T cells lacking TCR [58]. Importantly, transposons act more as non-viral gene addition tools rather than precise genome editors, as they integrate transgenes semi-randomly without targeted sequence modifications. While they bypass viral genome integration, their cellular delivery still depends on delivery methods like electroporation, AAVs or LNPs.

This distinction sets the stage for a more recent innovation in gene editing: CRISPR-associated transposases (CASTs) which are new classes of RNA-guided integrases that combine programmable CRISPR targeting with transposon-based DNA integration machinery. One of the most promising CAST platforms is HELIX, an engineered system (consisting of Cas12k, a nicking endonuclease–TnsB fusion and other components) that enables RNA-guided, DSB-free insertion of DNA cargos up to 10 kb with over 99% product purity, without dependency on HR. Though still in early development, HELIX has been functionally demonstrated in human HEK293T cells, where it achieved site-specific insertions into plasmid DNA with low efficiency, highlighting both its therapeutic promise and the need for further optimization [59]. In a parallel study, Lampe et al. achieved DSB-free, site-specific integration of large DNA cargos into the human genome with up to 5% efficiency using a different CAST variant [60]. In the most recent study, Liu et al. developed a simplified and compact CAST system that enables targeted insertion of therapeutic genes, like Factor IX into safe-harbor sites, with efficient performance across multiple human cell types [61]. Integra Therapeutics has developed a system called FiCAT (Find and Cut-And-Transfer), which combines CRISPR-Cas9 with the integration capacity of a modified piggyBac transposase to enable site-specific integration of large DNA fragments. Using reporter cargos (GFP, RFP, luciferase), as well as 9.5 kb FVIII gene, FiCAT achieved 5 – 22% on-target insertion *in vitro* (in HEK293T, K-562, and C2C12 cells) and targeted integration in 66% of treated mice *in vivo* at the safe-harbor locus, with stable expression sustained over several weeks. Notably, FiCAT was already shown to be compatible with non-viral delivery systems, including lipofection and JetPEI-based transfection, highlighting its translational potential [62].

Another example of a proprietary, next-generation gene editing platform emerging from industry is Tessera Therapeutics’ Gene Writing technology. The system reportedly harnesses reprogrammed retrotransposons and retrointegrases to insert or (re-)write genes at genomic loci defined by DNA-binding domain. The core mechanism appears to involve target-primed reverse transcription (TPRT), wherein a reverse transcriptase/endonuclease complex nicks the genomic DNA and reverse transcribes a co-delivered RNA template directly into the target site. These gene writers support precise, large-payload insertions, potentially spanning full gene coding sequences, surpassing the capabilities of base or prime editors [63,64].

A final emerging modality is the use of so-called bridge RNAs to guide locus-specific DNA recombination [65]. Discovered in prokaryotic IS110 elements, this system uses a structured non-coding RNA with two programmable loops that base-pair with both donor and target DNA, enabling a single recombinase to mediate insertion, excision, or inversion without DSBs or host repair pathways. The RNA-bridge system is highly programmable, relatively small, and requires only one protein and one RNA, offering a compact, modular alternative to CRISPR for seamless and brake-free rearrangements. However, its functionality has so far been demonstrated exclusively in prokaryotic systems, making it an intriguing prospect to watch as research explores its potential applicability in human cells.

### 2.2 Safety considerations across editing platforms

Beyond efficiency, the clinical translation of CRISPR-based immune cell engineering depends on a rigorous assessment of editing-associated genotoxicity. Each editor class carries a distinct risk profile that must be weighed against its therapeutic benefit.

Nuclease-based editors (Cas9, Cas12a) act through programmed DSBs, whose repair is intrinsically error-prone. In addition to the small indels generated by NHEJ, DSB repair can produce large deletions of several kilobases, complex genomic rearrangements at the cut site [32], and inter-chromosomal translocations. These translocations have been reported at ∼7% frequency between two simultaneous Cas9-induced edits in primary human T cells [66]. This is a particular concern for allogeneic CAR-T strategies that disrupt multiple loci simultaneously [38]. In rare cases, single DSBs can also trigger chromothripsis, large-scale chromosome shattering following micronucleus formation, observed in 4–7.5% of edited cells at clinically relevant loci [67]. DSBs further activate the MRN-ATM-p53 axis, driving p21-mediated apoptosis or senescence and selecting against cells with functional p53. This response is particularly consequential in long-lived stem cells, where it can reduce edited clone fitness and enrich for p53-deficient subpopulations [68,69]. Strategies designed to enhance HDR by inhibiting NHEJ, such as the use of DNA-PKcs inhibitor AZD7648, may amplify these on-target consequences, increasing kilobase-to megabase-scale deletions, chromosome arm loss, and translocations even at single target sites [70].

Base editors and prime editors substantially mitigate these DSB-driven risks by relying on nicking or template-directed mechanisms, and they consistently generate fewer translocations than nuclease editing in primary human T cells [37,44]. A recent head-to-head comparison demonstrated that quadruple ABE-edited allogeneic CAR-T cells outperform Cas9-nuclease-edited counterparts: translocations were detected exclusively in the Cas9-edited products, while ABE-edited cells showed reduced baseline p53 activation and superior *in vivo* antitumor efficacy [71]. However, base editors are not risk-free. They can introduce unintended DNA edits at genomic sites resembling the on-target sequence, bystander edits at nearby bases within the editing window, and transcriptome-wide RNA off-target mutations resulting from APOBEC1 (Cytidine Base Editor) or TadA adenine Base editor (ABE) deaminase activity [72]. Prime editors also carry their own residual risks, including small indels at the nick site and unintended edits templated by the pegRNA scaffold.

Note that delivery format independently shapes the safety profile and is discussed in detail in Section 3.

Importantly, these risks do not preclude therapeutic application of DSB-based editors. Cas9 and Cas12a remain important for applications requiring multiplex gene disruption or precise knock-in through HDR. Rather, the expanding understanding of editing-associated genotoxicity underscores the importance of rigorous quality control, including assays able to detect large deletions, chromosomal rearrangements, translocations, and aberrant DNA damage responses, particularly in multiplexed or clinically scaled manufacturing workflows.

CRISPR-based platforms enable diverse modes of genome and transcriptome manipulation. Cas9 and Cas12 nucleases induce site-specific double-strand breaks (DSBs) repaired by either template-free non-homologous end joining (NHEJ), generating small insertions or deletions, or homology-directed repair (HDR), which requires an exogenous donor DNA template for precise sequence changes. Base editors (BE) achieve single-nucleotide conversion without DSBs or donor templates. Prime editors (PE) encode the desired edit within the pegRNA itself, eliminating the need for a separate exogenous donor. RNA-targeting Cas13 effectors degrade transcripts, extending CRISPR functionality to post-transcriptional regulation. Catalytically inactive dCas9 fused to transcriptional effectors enables gene activation or repression without sequence alteration. CRISPR-associated transposases mediate site-specific donor DNA integration without DSBs. Together, these systems form a flexible toolkit spanning template-free (NHEJ, BE, Cas13, dCas9) and template-dependent (HDR, PE, transposases) modes of editing, regulating, and rewriting genetic information.

## 3 Challenges in CRISPR delivery

One of the persistent hurdles in translating these CRISPR-genome editors into clinical therapies is effective delivery. Specifically, how to introduce CRISPR components into target immune cells both efficiently and safely, while enabling precise genetic manipulation with minimal invasiveness. Immune cell types present distinct delivery challenges due to their hard-to-transfect nature and the need for clinical-scale manipulation [4]. Additionally, immune responses against Cas9 proteins, stemming from their prokaryotic origin, may present further challenges. The presence of antigen-reactive T cells against Cas9 was detected in majority of evaluated donors [73,74]. This issue may be particularly problematic for therapies requiring prolonged or repeated *in vivo* delivery of Cas9, as immune responses could lead to the elimination of Cas9-expressing cells, thereby compromising therapeutic efficacy. In contrast, preexisting adaptive immunity to Cas9 is less likely to impact *ex vivo* approaches using transient delivery formats such as mRNA or RNPs, since Cas9-derived peptides are only briefly presented on MHC class I molecules and are typically no longer detectable by the time the edited cells are reintroduced into the patient [73].

For all these reasons, the format in which CRISPR components are delivered plays an important role in determining both editing efficiency and safety, which is interconnected to different delivery modalities, including the vehicle or technique used, as well as whether the approach is applied *in vivo* or *ex vivo*.

### 3.1 CRISPR component delivery

**Fig. 2:**
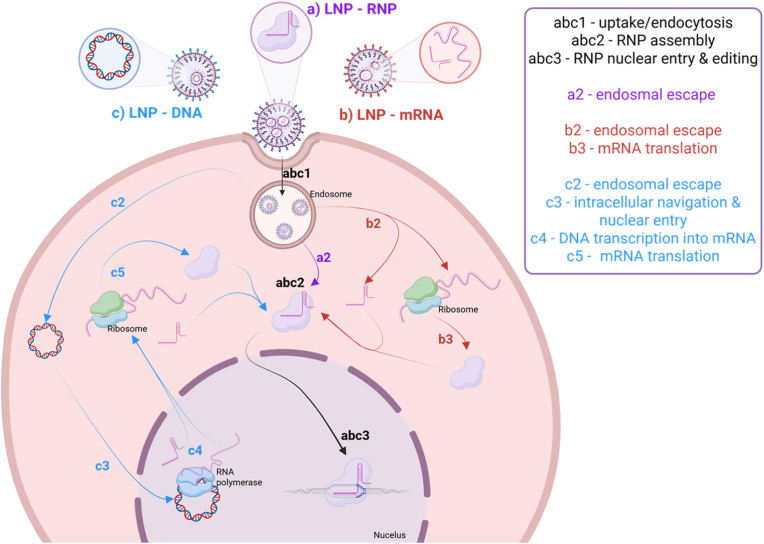
Intracellular processing of CRISPR-loaded lipid nanoparticles (LNPs) by cargo format.

CRISPR components: Cas9, sgRNAs as well as templates for recombination can be delivered in various formats, whether as plasmid DNA, messenger RNA (mRNA), or pre-formed ribonucleoprotein (RNP) complexes (Fig. 2). Each modality carries unique biochemical characteristics that determine its intracellular kinetics, immune recognition, persistence, and delivery requirements with its subsequent performance considerations.

#### 3.1.1 DNA

DNA is a foundational modality for CRISPR-Cas9 delivery due to its stability, ease of construction, and design flexibility, most delivered in the form of plasmid DNA (pDNA). Once inside the cell, pDNA must reach the nucleus for transcription of Cas9 and sgRNA, followed by translation in the cytoplasm and re-entry of the Cas9–sgRNA complex into the nucleus to perform genome editing. This multistep process introduces several bottlenecks, particularly nuclear trafficking and intracellular handling of the large DNA constructs (∼9.3 kb), which significantly reduce overall editing efficiency (Fig. 2). Moreover, prolonged or even stable Cas9 expression can increase the risk of off-target effects, genomic instability, and immunogenicity [75,76]. Despite these challenges, DNA-based systems offer distinct advantages. They support multiplexed gRNA expression from a single construct and allow the integration of cell-type- and time-specific promoters, providing a second layer of control. This tunable expression is especially beneficial in immune cell engineering, where tight regulation of Cas9 activity may enhance both safety and precision [77,78]. Beyond conventional plasmids, alternative DNA architectures, such as minicircle DNA (mcDNA) are being explored. By eliminating bacterial backbones and selection markers, mcDNA reduces vector size and may lower immunogenicity, making it an attractive option for delivering larger or more complex expression cassettes [75]. Additionally, in Cas13-based RNA editing systems, sustained expression from DNA vectors is not only efficient but often essential when long-term transcript regulation is required.

#### 3.1.2 mRNA

Another modality for delivering CRISPR-Cas9 components is mRNA (∼4.5 kb), offering rapid and transient gene editing capabilities. Upon cytosolic delivery, Cas9 mRNA is translated into Cas9 protein, which must be synchronously available with sgRNA to form an active complex and enable efficient gene editing. The transient expression profile of mRNA mitigates undesired off-target effects, reduces the risk of insertional mutagenesis, and lowers immunogenicity, which are the factors that collectively enhance the safety profile of this approach for therapeutic applications [11,75,79]. Moreover, mRNA issues related to its inherent instability and susceptibility to degradation by ribonucleases are overcome by coupling them with the appropriate delivery vehicle. Chemical modifications of RNA, such as incorporating N1-methylpseudouridine have been employed to enhance mRNA stability and efficiency, as well as to reduce immunogenicity (like in mRNA vaccines) [13,80,81]. However, recent studies (ours included) highlight that modifications are not always necessary, and that unmodified mRNA does not impair transfection efficiencies in immune cells (such as T cells, NK cells and B cells) which can be linked to the relatively low expression of RNA pattern recognition receptors (PRRs) and related adapter proteins in those cells [82,83]. Precise, cell-specific expression of mRNA can pose certain challenges, especially when delivered directly *in vivo*. Interestingly, an emerging avenue in mRNA-based therapies involves the regulation of mRNA expression through microRNA (miRNA) interactions. By incorporating miRNA target site into mRNA constructs, it is possible to avoid expression in off-target tissue/cells. For example, inclusion of miR-122 target sites in mRNA ensures that therapeutic proteins are not expressed in healthy hepatocytes, even if the therapeutic mRNA is inadvertently delivered there [84,85]. While this strategy holds promise, it remains under active investigation and has not yet been widely applied to Cas9 mRNA constructs.​ In summary, mRNA-based delivery of CRISPR-Cas9 components offers a balance of efficiency and safety.

Schematic representation of cellular uptake and intracellular trafficking of LNPs delivering CRISPR components as RNPs (a), mRNA (b), or DNA (c). Steps common to all modalities are shown as abc1 (uptake/endocytosis), abc2 (RNP assembly), and abc3 (nuclear entry and editing, when required). Format-specific pathways include a2 for endosomal escape of RNPs; b2 and b3 for mRNA endosomal escape and translation; and c2–c5 for DNA-specific processing, including endosomal escape, nuclear entry, transcription, and mRNA translation.

#### 3.1.3 RNP

Direct delivery of RNP, composed of Cas9 protein pre-complexed with sgRNA, represents the most rapid CRISPR-Cas9 format and allows fast, efficient, and transient genome editing. The short-lived intracellular presence minimizes off-target effects, cytotoxicity, and immunogenicity, while eliminating the risk of genomic integration. Moreover, when Cas9 is delivered as RNP, preexisting adaptive immunity is unlikely to hinder *ex vivo* therapies, as Cas9 peptides are just transiently presented on MHC I, which reduces the risk of elimination of (re-)introduced cells. RNPs are widely used in immune cell engineering for precise editing, which is improved by co-delivery with HDR templates carrying truncated Cas9 target sequences that help guide the template to the nucleus through interaction with the RNP complex [75,86]. Even with large size of Cas9-sgRNA RNP (∼190 kDa), the complex is overall negatively charged, which facilitates and opens delivery opportunities with electroporation (EP) and/or cationic NPs [87]. Moreover, RNP delivery offers a robust platform for cells with low transcription and translation activity, required for efficient editing from nucleic acid-based platforms, such as non-activated T cells [88,89]. However, standard RNPs lack intrinsic regulatory mechanisms to modulate their activity post-delivery, unlike DNA-based methods that utilize promoter sequences for gene expression control. To achieve spatiotemporal control over CRISPR activity, researchers have developed photocaged sgRNAs that inhibit RNP-DNA interactions until exposure to UV light restores base-pairing, enabling light-activated genome editing [90]. Additionally, as highlighted in the review by Zhuo et al., other ways of spatiotemporal control, such as small molecules are being explored as regulatory switches that can modulate the activity of gene editors [78].

### 3.2 Conventional CRISPR delivery techniques

**Fig. 3:**
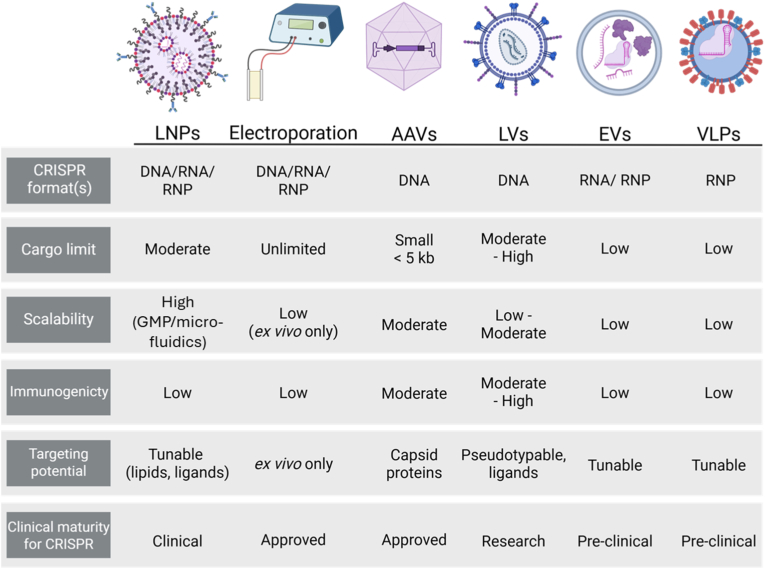
Comparison of delivery platforms for CRISPR cargo formats.

The format of CRISPR components has an essential implication on delivery approaches, which remains a persistent challenge for translating genome editors into viable therapies. Still, numerous options are possible: both viral and non-viral (Fig. 3).

#### 3.2.1 Adeno-associated viruses (AAVs)

Adeno-associated viruses (AAVs) are among the most widely used delivery vectors for *in vivo* genome editing. These small, non-enveloped ssDNA viruses are favored for their low immunogenicity, lack of pathogenicity, and broad tropism – key attributes that make them well-suited for gene therapy applications. AAVs typically remain episomal and do not integrate into the host genome under normal conditions, except occasionally at the AAVS1 safe harbor locus [9,91]. However, the limited packaging capacity (∼4.7 kb) poses a major constraint, often requiring dual-vector systems to deliver Cas9 and sgRNAs separately, which complicates synchronization and reduces efficiency. Strategies to overcome this include usage of dual AAVs where one vector encodes Cas9 and the other carries the sgRNA and donor template [91]. Alternative approaches include employing smaller Cas variants (such as SaCas9 or CasX) that are more compatible with AAV packaging constraints [92]. AAVs can transduce both dividing and non-dividing cells, and through the use of different serotypes and capsid variants, they can be engineered to target specific immune cells and hematopoietic stem cells for immune-modulating applications. Those vectors have been explored in several immune cell contexts, such as CAR-cell generation and knock-ins in T and NK cells, as well as gene correction in HSCs; however, their performance in primary immune cells remains variable [[93], [94], [95], [96], [97]]. Moreover, pre-existing immunity to AAV capsids and the inflammatory responses observed at higher doses pose challenges to repeated administration and systemic delivery. Current research is focused on engineering next-generation AAV capsids with enhanced cell/tissue specificity, immune evasion properties, and expanded cargo capacity, aiming to expand their utility for both *in vivo* and *ex vivo* CRISPR applications in immunotherapies [98]. Despite clinical success and broad application of AAVs, their production, scale-up, and costs of AAV-based therapies remain major practical obstacles [99].

#### 3.2.2 Lentiviruses (LVs)

Lentiviruses (LVs) are RNA-based viruses that offer high transduction efficiency and a large cargo capacity (∼8 kb), allowing the co-delivery of Cas proteins and multiple sgRNAs in a single vector [91]. Importantly, they exhibit low-to-moderate immunogenicity, and they can transduce wide range of both dividing and non-dividing cells efficiently. Targeting specificity can be enhanced either through pseudotyping (using glycoproteins from other enveloped viruses) or by incorporating targeting antibodies directly into the vector particles. These features have led to widespread use of LVs in hematopoietic and immune cells (T cells, B cells, NK cells, DCs …) [[100], [101], [102], [103], [104]]. LVs were employed in first approved CAR-T cell therapy, Kymriah [105]. They integrate into the host genome following reverse transcription, enabling stable gene expression and transmission to daughter cells after mitosis. A major concern with LV use is the potential for genomic integration that can disrupt host genes or activate oncogenes, thereby increasing the risk of insertional mutagenesis. This risk is further amplified when combined with CRISPR-Cas9, which introduces DNA double-strand breaks and may contribute to additional genomic instability [91]. To mitigate these risks, non-integrating LVs (integration-deficient) have been developed. They enable transient-to-sustained episomal expression, thus increasing the safety profile and offering new avenues for the employment of this approach for CRISPR delivery [106,107]. Nonetheless, the cost and complexity associated with the production of clinical-grade lentiviral vectors remain significant considerations for therapeutic applications [99].

#### 3.2.3 Electroporation (EP)

In contrast to viral vectors, non-viral physical delivery methods, especially electroporation, have become a gold standard in *ex vivo* editing protocols for CRISPR engineering of immune cells. EP uses short, high-voltage pulses to transiently permeabilize cell membranes, enabling direct cytosolic entry of charged biomolecules such as plasmid DNA, mRNA, or pre-assembled RNP complexes, offering rapid, carrier-free and efficient delivery, avoiding vector-related immunogenicity [75,108]. These features have led to its broad use in immunotherapies, including clinical-grade T cell engineering [28,86]. Importantly, co-delivery strategies combining EP of Cas9 RNPs with AAV6 vectors encoding donor templates have demonstrated high-efficiency targeted integration into safe harbor loci (such as AAVS1), enhancing homology-directed repair (HDR) outcomes in primary immune cells [[109], [110], [111], [112]]. However, despite these promising results, EP comes with significant shortcomings. The application of electric pulses can compromise cell viability, impair proliferation and mitochondrial function, and alter cellular phenotypes – effects that are especially concerning in clinical-grade, sensitive immune cell populations [[113], [114], [115], [116]]. Successful outcomes are highly dependent on finely optimized cell-type-specific protocols, including voltage, pulse width, and buffer composition, all of which contribute to the complexity and the cost of EP-mediated therapies [75]. Moreover, direct *in vivo* electroporation remains largely unfeasible and unscalable in clinical contexts, although promising results have been demonstrated in animal models. For example, one study used a conductive hydrogel-based system to deliver CRISPR-Cas9 DNA into T cells within lymph nodes, generating PD-1-deficient T cells that suppress tumor growth and recurrence in melanoma models [117].

#### 3.2.4 Extracellular vesicles (EVs)

Beyond traditional viral and non-viral vectors, emerging modalities such as extracellular vesicles (EVs) and virus-like particles (VLPs) are gaining attention for their potential in delivering CRISPR machinery to immune cells.​ EVs are cell-derived membranous nanoparticles capable of transferring proteins and nucleic acids between cells, making them promising candidates for CRISPR-Cas9 delivery due to their low immunogenicity, high biocompatibility and *in vivo* stability. Strategies for EV-mediated delivery include passive loading through overexpression of CRISPR components, active loading via protein or RNA dimerization, and post-purification loading techniques [118]. These approaches have shown promise for both *in vitro* and *in vivo* gene editing, with EVs offering the added advantage of immune cell-specific targeting through surface protein engineering or the incorporation of targeting ligands [119,120]. However, limited delivery efficiency, lack of HDR template co-delivery strategies, along with unresolved issues in scalability and regulatory compliance, currently hinder the therapeutic translation of EV-based CRISPR systems [121].

#### 3.2.5 Virus-**like** particles (VLPs)

VLPs are engineered, replication-incompetent particles that can encapsulate and deliver functional proteins and nucleic acids into target cells, which makes them a promising candidate for delivery of gene editors to immune cells. By fusing Cas9 to viral structural proteins, such as Gag, these particles can efficiently package the Cas9 protein complexed with sgRNAs.​ A notable example is the development of “nanoblades”, retroviral-VLPs which have demonstrated high efficiency in gene editing across various human immune cells, including T cells, B cells, and HSPCs after pseudotyping with envelope glycoproteins from baboon endogenous virus [122]. In another example, lentiviral Cas9-VLPs have demonstrated efficient and multiplexed genome editing in primary human T cells, enabling simultaneous CAR integration and KO of allogeneic targets. Pseudotyping with specific viral glycoproteins (e.g., HIV-1 Env) allows selective editing of CD4^+^ T cells, highlighting their potential for cell-type-specific *in vivo* genome engineering [123]. This potential was exemplified by recent advances. Ling et al. developed a modular system known as RIDE, which uses engineered lentiviral components and MS2-tagged guide RNAs to package and deliver Cas9 directly into target cells. By customizing the VLP surface with specific glycoproteins, such as DC-SIGN- and CD3-recognizing envelopes, they achieved efficient and selective editing in dendritic cells and T cells, respectively. Impressively, RIDE could efficiently edit the huntingtin gene in patients’ induced pluripotent stem cell-derived neurons and was tolerated in non-human primates [124]. In parallel, Hamilton et al. introduced enveloped delivery vehicles (EDVs) that rely on antibody-derived targeting: single-chain fragments (scFvs) on the particle surface to direct delivery toward human immune cells, CD4^+^ and CD8^+^ T cells. This approach enables high-efficiency genome editing, even in resting T cells, and supports *in vivo* generation of functional CAR-T cells [125]. Despite their promising precision and versatility, Cas-VLPs remain largely experimental, without clinical validation and scalability, prompting ongoing efforts to improve targeting, yield, and production toward clinical translation.

Overview of key delivery modalities used for CRISPR genome editing, including lipid nanoparticles (LNPs), electroporation, adeno-associated viruses (AAVs), lentiviral vectors (LVs), extracellular vesicles (EVs), and virus-like particles (VLPs). Parameters compared include cargo format, cargo limit, scalability, immunogenicity, targeting potential, and clinical maturity. Notably, LNPs exhibit high scalability in the context of GMP-compliant manufacturing, enabled by reproducible microfluidic formulation processes that allow controlled particle size distribution, encapsulation efficiency, and robust batch-to-batch consistency, as demonstrated in large-scale deployment of clinically approved mRNA-LNP therapeutics.

## 4 Lipid nanoparticles as gamechangers in delivering gene editors

Despite the promise of the mentioned delivery systems, there remains an imperative need for more efficient, safe, and scalable delivery methods that combine the high specificity profile of virus (-like) particles with low-immunogenicity profile and high-efficiency of non-viral methods. Lipid nanoparticles (LNPs) have emerged as a compelling alternative, and with their global application on billions of people in COVID-19 vaccine through several injections, they have showcased their advantages of unmatched biocompatibility, tunability, and manufacturability. The transient, non-integrating nature makes them particularly attractive for gene editing applications where permanent genomic changes are achieved without prolonged nuclease expression. Recent studies have shown that CRISPR-Cas9 mRNA/LNP formulations can achieve efficient editing in primary human T cells while dramatically preserving cell viability and functionalities compared to EP-based approaches [116]. Moreover, LNPs have been utilized to safely and efficiently deliver RNP forms of base and prime editors *in vivo*, achieving therapeutic correction with high precision and minimal off-target effects [126]. While preclinical successes have been encouraging, a true translational leap was demonstrated in a recent case study where LNPs enabled effective *in vivo* base editing in a real-world pediatric patient. It was proposed to treat severe carbamoyl-phosphate synthetase 1 deficiency, a disease with an estimated 50% mortality in early infancy. This marked the first time a bespoke CRISPR therapy moved from diagnosis to treatment in under a year, with the treatment developed and given within months, helping the baby improve and grow despite a life-threatening condition [127]. Collectively, these advances highlight the emergence of LNPs as versatile enablers of the full CRISPR toolbox, capable of delivering diverse editing modalities into cells both *ex vivo* and *in vivo*, with precision, scalability, and clinical promise. Therefore, the following section explores how their tailored molecular design supports CRISPR-Cas delivery for immune engineering.

### 4.1 Molecular architecture and composition of LNPs

Efficient encapsulation and delivery properties of LNPs (∼100 nm) come down to a smart molecular architecture of four main components: an ionizable lipid, a sterol (cholesterol), a helper phospholipid, and a PEGylated lipid, most commonly at the molar ratio of 50-38.5-10-1.5 (reviewed in work by Haque et al. [128]). Each component and its prevalence contribute to distinct physicochemical and functional properties [129].

Alongside composition, the method of formulation represents a key parameter influencing nanoparticle properties. Rapid mixing of lipids in ethanol with the aqueous RNA phase typically under acidic conditions drives spontaneous self-assembly into particles with defined size, high encapsulation efficiency, and narrow polydispersity. Microfluidic platforms such as staggered herringbone micromixers offer millisecond-scale mixing and excellent reproducibility, while simpler bulk methods like T-junction mixing, though less precise, remain attractive for their scalability in larger-volume manufacturing [130]. LNPs can thus be prepared through a range of approaches, but the choice of method has a direct impact on their structural attributes and ultimately on biological performance [131,132].

#### 4.1.1 Ionizable lipids

Ionizable lipids are the engine of LNP functionality, condensing negatively charged cargos (plasmid DNA, mRNA or Cas9-RNP) at acidic pH during formulation, while remaining largely neutral at physiological pH, after buffer exchange, to reduce toxicity and off-target uptake [133]. Then, upon cellular internalization and endosomal acidification, these lipids become protonated, enabling fusion with the endosomal membrane and cargo release into the cytosol [134,135]. Factors such as ratio of ionizable lipid amines to nucleic acid phosphates (N/P ratio), lipid tail branching, pKa tuning, and inclusion of biodegradable bonds (e.g. esters or disulfides) in ionizable lipids significantly impact LNP stability, cargo loading, *in vivo* distribution and protein corona formation, along with intracellular processing [[136], [137], [138], [139]]. Due to this, strategic refinements and optimizations are necessary to achieve enhanced and tailored cell- and purpose-specific applicability. Notably, biodegradability of ionizable lipids increases transfection efficiency while minimizing toxicity, which can eventually enhance CRISPR-Cas9 efficiency, making repeated dosing both safe and feasible [140,141]. Currently, FDA-approved ionizable lipids such as ALC-0315 (used in BNT162b2), SM-102 (used in mRNA-1273), and DLin-MC3-DMA (used in Onpattro) have become the clinical benchmarks for RNA delivery, and are frequently employed in preclinical studies targeting immune cells due to their well-characterized safety, efficacy, and manufacturability. However, given the heterogeneity of immune cell types and therapeutic contexts, a *one-fits-all* approach remains suboptimal, which eventually drives the development of next-generation ionizable lipids through rational design, high-throughput screening, and structure-activity relationship studies aimed at enhancing potency, specificity, and biodegradability [142]. Interestingly, piperazine-containing ionizable lipids (Pi-Lipids) identified through high-throughput *in vivo* barcoding of 65 chemically distinct LNP variants, have been shown to preferentially deliver mRNA to immune cells *in vivo* across 14 cell types without targeting ligands [143]. Similarly, C14-4 has emerged as a top-performing ionizable lipid for primary T cell transfection, outperforming FDA-approved benchmarks with lower cytotoxicity [144,145].

#### 4.1.2 Sterols

Cholesterol and its analogues serve as a structural stabilizer and modulator of lipid packing and membrane fusion, bridging the LNP core and lipid headgroups [133,146]. Notably, Patel et al. demonstrated that incorporating naturally occurring cholesterol analogues (e.g. β-sitosterol) into LNPs can induce non-spherical morphologies and enhance endosomal escape, leading to significantly improved intracellular mRNA delivery [147]. Further, substituting 25–50% of standard cholesterol with 7α-hydroxycholesterol in LNP formulations led to up to a twofold increase in mRNA delivery to primary human T cells. This improvement was attributed to altered endosomal trafficking and reduced recycling, highlighting a promising strategy for targeted T cell delivery [148]. Another effective approach involved the replacement of cholesterol with a derivative containing a pH-sensitive imidazole group, which facilitated endosomal escape and improved overall delivery efficacy [149]. Together, these strategies underscore the potential of cholesterol analogue modification to optimize LNP performance for enhanced mRNA delivery.

#### 4.1.3 Helper lipids

Helper lipids such as DSPC (distearoylphosphatidylcholine) or DOPE (1,2-Dioleoyl-sn-glycero-3-phosphoethanolamine) support LNP structure, promote endosomal destabilization, and even determine the biodistribution fate of the particles, depending on their geometry, saturation and charge [146,150]. Notably, replacing standard helper lipids with charged alternatives enables selective mRNA delivery to specific immune and epithelial cell populations, where cationic lipids like DOTAP enhance transfection of epithelial cells, and anionic lipids such as phosphatidylserine (PS) favor uptake by myeloid and lymphoid cells through charge modulation and scavenger receptor-mediated endocytosis. These tunable properties make helper lipids versatile tool for guiding LNP biodistribution and enhancing therapeutic delivery to specific cell types [151].

#### 4.1.4 PEG-lipids

PEG-lipids constitute the outermost layer of LNPs, forming a hydrophilic corona, and are critical for controlling LNP size, preventing aggregation, opsonization and prolonging circulation time [137,152]. However, PEG must be carefully balanced, as excessive PEGylation can reduce cellular uptake by sterically hindering interactions between the nanoparticle surface and the cell membrane [153]. The anchoring strength of PEG-lipids, dictated by alkyl chain length, further shapes LNP behavior: short-chain PEGs (e.g., C14 DMG-PEG) rapidly shed, promoting protein corona formation and enhancing transfection; whereas long-chain PEGs (e.g., C18 DSG-PEG) prolong circulation, but may impair delivery efficiency. Thus, fine-tuning PEG shedding is crucial to balance stealth with effective cellular delivery [154,155].

As a practical design rule, due to the rapid shedding of DMG-PEG (C14) the steric barrier is removed prior to cellular contact, facilitating transfection – a property not shared by stably anchored C18 variants [156,157]. The relationship between anchor length and biodistribution is however not straightforward: C18 DSG-PEG at reduced molar ratios (0.75 mol%) has been shown to redirect LNP accumulation toward the spleen and activate splenic dendritic cells and CD4^+^ T cell populations *in vivo* in a cancer vaccine context, suggesting that anchor length and molar ratio act as orthogonal and potentially exploitable levers for immune organ targeting [158]. Whether analogous formulation strategies can be translated to *in vivo* immune cell engineering remains to be established, and cell-type-specific PEG-lipid design rules for immune populations remain largely underexplored.

PEGylation comes with certain immunological trade-offs as repeated dosing can trigger anti-PEG IgM and IgG responses, leading to the accelerated blood clearance (ABC) effect, reduced efficacy, and in some cases, hypersensitivity or even anaphylaxis. Collectively, these delivery trade-offs are known as the PEG dilemma [153,159].

This is particularly consequential for *in vivo* immune cell engineering requiring repeated dosing: ABC-driven clearance can reduce second-dose expression efficacy by 15-55% depending on PEG-lipid structure, with losses compounding across further administrations [160]. Among emerging alternatives, polysarcosine (pSar) provides equivalent steric stabilization to PEG while eliciting negligible anti-pSar antibody responses and reduced complement activation [161]. Critically, direct substitution of PEG with pSar lipids retains or enhances mRNA transfection efficiency depending on ionizable lipid identity, with pSar LNPs outperforming PEG counterparts by up to ∼40 percentage points in immune-relevant cell types at equivalent doses [162,163]. Like PEG, pSar chain length and lipid tail structure govern the circulation-transfection trade-off, offering equivalent formulation tunability without the immunogenic liability [164].

#### 4.1.5 Beyond “classical” LNPs

Ultimately, the precise molar ratios and molecular designs of these four components dictate LNP behavior, affecting everything from immune cell uptake to genome-editing efficacy. Several rational modifications to standard LNP composition have shown promise in overcoming delivery barriers to immune cells. One approach is to introduce a fifth functional lipid, such as in Selective Organ Targeting (SORT) approach or through the addition of bioactive phospholipids, which can fine-tune surface charge, modulate the protein corona, redirect cellular tropism, and consequently impact transfection efficiency and cytokine responses in immune cells [165,166]. Complementing these strategies, a recent study showed that removing both phospholipids and cholesterol still supports efficient mRNA delivery, challenging long-held design assumptions. This three-component system highlights how re-engineering core lipids can enhance functionality and broaden delivery to non-hepatic cells [167].

Except for lipid identity, modulating lipid molar ratios beyond conventional approach (50-38.5-10-1.5), has shown promise to significantly influence LNP structural properties, biodistribution and pharmacokinetic properties, as well as transfection efficiency. For instance, LNPs incorporating higher proportions of helper lipids such as egg sphingomyelin (ESM) have been shown to increase protein expression in hepatic and extrahepatic tissues, including spleen and bone marrow, and to promote stronger interactions with myeloid cells such as monocytes and macrophages [168,169]. More recently, this was extended to liposomal LNPs with equimolar ESM and cholesterol (with only 20% of ionizable lipid), which further enhanced transfection in lymphoid tissues, likely due to the prolonged circulation (up to 15-fold increase when compared to conventional formulations) [170].

These adjustments not only influence LNP structure and stability but may also shift tropism toward key immune cell populations, offering a formulation-driven route to optimize CRISPR-Cas delivery in immunotherapy. Collectively, these rational design modifications hold promises for enabling precise, high-efficiency CRISPR-Cas delivery to immune cells, in both *ex vivo* and *in vivo* environments.

### 4.2 Enabling immune cell *in vivo* targeting with LNPs

**Fig. 4:**
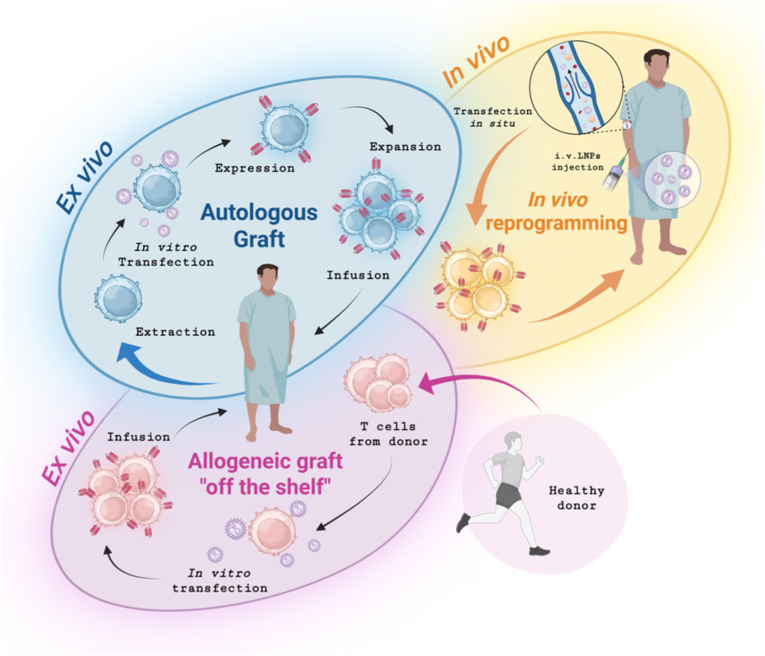
Strategies for nanoparticle-mediated payload delivery in cell immunotherapy.

*In vivo* targeting immune cells provides significant advantages over conventional *ex vivo* approaches by avoiding the labor-intensive processes associated with cell isolation, manipulation, and reinfusion (Fig. 4). This approach not only reduces manufacturing complexity, associated costs, and timelines but also maintains essential immune cell interactions within their native physiological contexts, thereby improving therapeutic consistency and minimizing variability, allowing broader potential across diverse clinical settings.

Up to date, most of mRNA-based drugs are vaccines, which primarily use intramuscular (i.m.) injections, enabling local antigen expression at injection sites and secondary lymphoid organs [[171], [172], [173]]. However, future gene editing LNP-based therapies will face challenges regarding their administration routes, given that the payload will need to be delivered specifically to the organ or cells of interest to precisely treat the disease at genomic level. This represents a challenge knowing that most of the current LNP-based systems exhibit a natural tropism for the liver, largely due to their physicochemical properties, including size, lipid composition, and the presence of cholesterol, which promote ApoE enrichment in the protein corona [[174], [175], [176]]. Therefore, various approaches to achieve delivery beyond the liver, targeting immune cells are critical.

This section will summarize different strategies that could be leveraged to achieve immune cell targeting based on three different levels: (i) at the administration route, (ii) lipid-shell or (iii) cargo/mRNA-sequence level to achieve cell-specific extra-hepatic delivery.

The schematic contrasts *ex vivo* and *in vivo* approaches for CAR-T cell generation. In the *ex vivo* setting (left), immune cells, either autologous or allogeneic, are isolated, transfected with therapeutic payloads (e.g., mRNA via LNPs), expanded, and reinfused. Allogeneic T cells require prior engineering using CRISPR–Cas systems to disrupt endogenous TCR and/or HLA molecules and ensure compatibility. In contrast, *in vivo* strategies (right) rely on direct systemic or local administration of targeted nanoparticles to transfect immune cells within the patient, enabling cell engineering without cell extraction or expansion.

#### 4.2.1 Routes of administration

**Fig. 5:**
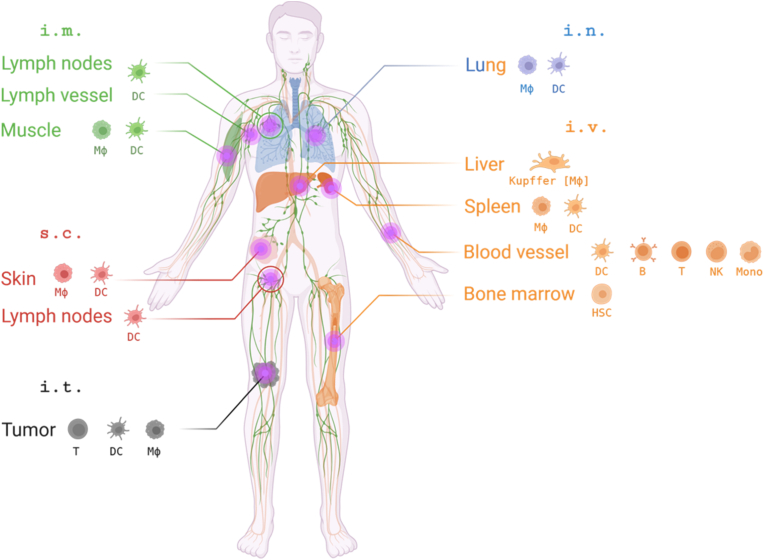
**Schematic mapping of relevant organs and immune cell types targeted by different administration routes:** intravenous (i.v.), intramuscular (i.m.), subcutaneous (s.c.), intratumoral (i.t.), and intranasal (i.n.). Each route provides access to distinct tissues and immune compartments, allowing for tailored immunotherapeutic strategies. Notably, the lungs are accessible via both i.v. and i.n. routes, engaging resident macrophages (MΦ) and dendritic cells (DC) through distinct mechanisms; this dual accessibility is reflected in the figure by the combined color annotation for the lung label. Targeted immune cells include dendritic cells (DC), macrophages (MΦ), T cells, B cells, natural killer (NK) cells, hematopoietic stem cells (HSC), and monocytes (Mono). These delivery routes facilitate selective engagement of immune subsets, supporting optimized therapeutic outcomes through route-specific targeting. (For interpretation of the references to color in this figure legend, the reader is referred to the Web version of this article.)

Administration routes significantly influence LNP biodistribution and targeting efficacy. Intravenous (i.v.) injections effectively target circulating immune subsets such as T cells, B cells, NK cells, and monocytes (Fig. 5). In contrast, tissue-resident cells like dendritic cells (DCs) and macrophages require more specialized, localized approaches [2,177,178]. Although intramuscular (i.m.) injections enable direct transport of LNPs to lymph nodes, this route of administration is now rather used for the treatment of muscle pathology, such as Duchenne muscular dystrophy (DMD) [173]. Kenjo et al. demonstrated the utility of repeated i.m. injections with low-immunogenicity CRISPR-Cas9 LNPs for exon skipping in DMD models [11].

Alternative routes such as subcutaneous (s.c), intratumoral (i.t.), and intranasal (i.n.) have also been explored. For instance, a recent study from Mao et al. demonstrated that intra-tumoral injection of LNPs with low cholesterol density enhance mRNA uptake, endosomal escape, and CRISPR-Cas9 delivery, leading to efficient PD-L1 KO in conventional DCs [179]. Another study from Wang et al. used a dissolvable microneedle transdermal patch to co-deliver polymer-encapsulated Cas9 RNPs and a glucocorticoid (Dexamethasone) into keratinocytes and dendritic cells, leading to disruption of the NLRP3 inflammasome *in vivo*; and reducing inflammation in mouse models of psoriasis and atopic dermatitis [180]. Recently, nebulization has also shown to be of interest for lung targeting and precise CRISPR-Cas9 delivery [181]. Aerosolized delivery, demonstrated by Huang et al., enabled precise CRISPR-Cas9 delivery to alveolar macrophages with minimal off-target effects [182].

Among the various administration routes, i.v. delivery remains the most extensively explored for targeting circulating cells and specialized organs such as spleen. In a study by Hamilton et al., high-throughput screening using molecular barcoding technology revealed fundamental differences between i.m. and i.v. LNP administration for immune cell targeting, especially for targeting monocytes and DCs. For i.v. injection, ionizable lipid structure emerged as the primary determinant of immune cell uptake, contrasting with i.m. administration where excipient molar composition played a more dominant role in influencing performance [183].

This differential distribution based on composition was also observed at the organ level in a study published by Cheng et al., showing that adding a supplemental “SORT” (Selective Organ Targeting) lipid such as DOTAP, DDAB, EPC, and 18 PA to an otherwise liver-directed LNP can retarget its organ tropism and allow efficient gene editing application [165]. Later, Dilliard et al. found that the ability of SORT LNPs to induce liver, splenic or lung-specific gene editing hinges on how the structure of quaternary ammonium lipids shapes the protein corona, modulating opsonization and subsequent biodistribution [184]. By combining dual SORT lipid, Kim et al. were able to deliver base editor mRNA and sgRNA for simultaneous correction in the liver and lungs, directly addressing the multi-organ pathology of Alpha-1 antitrypsin deficiency [185].

ApoA1-inspired nanoparticles (aNPs), developed by Hofstraat et al., leverage natural protein corona interactions, effectively targeting myeloid cells and hematopoietic stem and progenitor cells in bone marrow to deliver nucleic acids (siRNA, antisense oligonucleotides, and mRNA) [186]. As previously mentioned, long-circulating LNPs containing bilayer-forming lipids such as ESM and Chol exhibited enhanced extrahepatic delivery by minimizing opsonization and extending circulation half-life [187].

#### 4.2.2 Active targeting: conjugation of ligands to achieve specificity

**Fig. 6:**
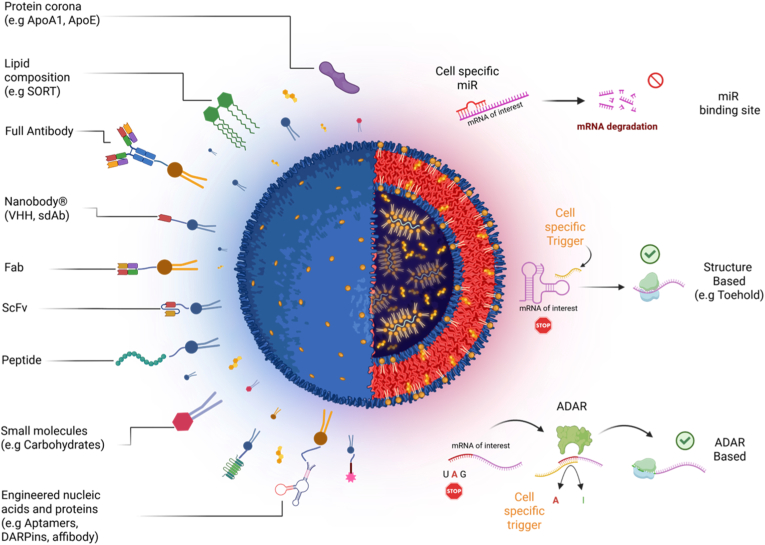
Strategies for enhancing cell-specific targeting and translational control via LNP-mRNA delivery.

Surface modification of LNPs allows the engrafting of many disposable targeting moieties, each offering distinct advantages in terms of affinity, ease of handling, stability, and biodistribution (Fig. 6).

##### 4.2.2.1 Antibodies and antibody fragments

Antibodies and their derivatives including fragments, such as Fab’, scFv, F(ab’)_2_, and nanobodies® (VHH), offer high specificity and affinity for cell surface antigens. Full-length antibodies (∼150 kDa) are widely used to target various immune cell populations. For instance, anti-CD3, CD4 and CD5 antibodies have been used to target T cells [[188], [189], [190], [191]], anti-CD117 for hematopoietic stem and progenitor cells (HSPCs) [192], anti-Ly6C for inflammatory leukocytes [193], and anti-F4/80 for macrophages [194]. However, the large size of antibody can hinder tissue penetration and quickly saturate the LNP surface limiting the number of targeting ligands per particle [195,196]. Antibody fragments such as Fab’ and scFv improve tissue access and allow higher ligand density [[197], [198], [199]]. Singh et al. found that although full-length anti-CD4 antibody alone exhibited higher binding to CD4-high T cells compared to Fab' alone due to greater avidity, Fab'-nanoparticle conjugates (Fab'-NCs) achieved comparable targeted delivery efficiency to full antibody-nanoparticle conjugates. Fab'-NCs proved more potent in targeted drug delivery to CD4-high T cells. This was attributed to the smaller size of Fab' fragments, which allowed a greater number of ligands per nanoparticle. Moreover, Fab' fragment provides a site-selective conjugation strategy via its intact thiol moiety, offering a more controlled and reproducible modification, which contrasts with the typically heterogeneous modifications often seen with full-length antibodies [200]. Smaller antibody formats, such as scFvs have also been explored. DEC205 ScFv-LNPs have been used to target dendritic cells to deliver siRNA [201], anti-CD19 liposomes enhance B cell delivery of doxorubicin [202], and anti-CD22 ScFv has shown potentiality as alternative for B cell targeting [203]. ScFvs are also used in ASSET-LNPs (Anchored Secondary ScFv Enabling Targeting) [204], a modular system that binds the Fc domain of antibodies to direct LNPs to T, B, and monocyte subsets [193,205]. Dietmair et al. introduced bispecific antibodies (BsAbs) linking LNPs to cells via dual scFvs, one for PEG-lipid and the other binding the cell surface receptor as EGFR. In their “pre-targeting" strategy, BsAbs first bind to target cells, followed by LNP administration. This approach enhanced specificity and resulted in over 7-fold improved tumor delivery of mRNA while reducing off-target delivery to the liver and spleen, compared to conventional pre-mixed BsAb-LNP systems [206]. Nanobodies® also known as single single-domain antibodies (SdAb or VHH, ∼12–15 kDa) retain full antigen specificity with high solubility [207]. Though less reported, they show promise: Bouma et al. used VHH-liposomes to target CD169^+^ macrophages and DC-SIGN^+^ dendritic cells for cancer vaccines [208], while Sawyer et al. achieved ∼80% CAR expression in CD8^+^ T cells using CD8-specific nanobody-LNPs *in vitro* and in humanized mice [209]. Similarly to ASSET strategy, Chen et al. used anti-Fc nanobodies® on LNPs for antibody capture and T cell targeting [210].

##### 4.2.2.2 Peptides

Peptides (∼1–5 kDa), whether naturally derived or synthetically engineered, offer advantages as targeting ligands due to their easy chemical modification and efficient conjugation, with favorable tissue penetration and generally lower immunogenicity compared to antibodies. They can remain stable under harsh conditions including dry powder formulations [211]. However, due to their lower binding affinity and rapid clearance, multivalent display is often needed for efficient targeting [211,212]. Swart et al. used a tripeptide (Leu-Asp-Val) targeting VLA-4 to deliver siRNA-loaded LNPs to bone marrow, notably to immature hematopoietic progenitors [213]. Polito et al. designed liposomes bearing an HPV antigen and a dendritic cell-targeting peptide to enhance vaccine efficacy [214], while Zhang et al. formulated DP7-C (cholesterol-modified peptide) liposomes to promote DC maturation and antigen presentation [215]. For T cells, Endsley et al. developed CD4-binding peptide–liposomes that selectively target CD4^+^ T cells [216]. Interestingly, Papp et al. used a CD47-derived peptide to engage SIRPα on phagocytes, reducing clearance by acting as a “don't eat me” signal. When combined with anti-CD4, CD31, or CD117 antibodies, this strategy achieved selective delivery to T cells, endothelial cells, and HSCs, with minimal off-target effects [217].

##### 4.2.2.3 Small molecules ligands

Small molecule ligands, such as carbohydrates and other chemical moieties, offer synthetic versatility, ease of production, and control. Folate-modified LNPs have been used to target activated macrophages [218]. Among these ligands, mannosylated lipids are widely employed. Lei et al. developed monosaccharide-conjugated lipids (Man-, Glu-, and Gal-lipids) and showed that mannosylated LNPs achieved the highest mRNA delivery to dendritic cells *in vivo* after i.m. injection via mannose receptor targeting [219]. Similarly, Mahalingam et al. used a shikimic acid–conjugated lipid (a glycomimetic) to enhance mRNA vaccine uptake by DCs and macrophages [220]. Tuning carbohydrate number and orientation can enhance avidity for C-type lectin receptors like DC-SIGN and mannose receptor, facilitating APC targeting [221,222]. Beyond mannose receptors, Siglecs (sialic acid-binding immunoglobulin-like lectins) offer highly specific immune cell targets [223]. Using glycan-modified liposomes, Chen et al. achieved doxorubicin delivery to B lymphoma cells via CD22,[224] while Srivastava et al. leveraged the same axis to induce B cell tolerance [225].

##### 4.2.2.4 Aptamers

Aptamers are short single-stranded nucleic acid ligands, typically 20 to 100 nucleotides in length (approximately 5–30 kDa) and composed of DNA, RNA, or chemically modified oligonucleotides. They fold into unique three-dimensional structures that enable high specificity and affinity for a wide range of targets, including proteins, small molecules, and cell receptors. They offer deep tissue penetration, low immunogenicity, chemical versatility and high binding affinity due to their 3D folding, making them attractive tools for targeted delivery. However, unmodified aptamers are often prone to nuclease degradation in biological fluids, which can limit their *in vivo* stability [[226], [227], [228], [229]]. Aptamers can be readily conjugated to LNPs. Huang et al. used RNA aptamers to direct drug-loaded LNPs into circulating monocytes, demonstrating therapeutic efficacy in models of heart disease and pancreatic cancer [230]. Similarly, Rajagopalan et al. developed a bispecific conjugate combining a 4-1BB-specific DNA aptamer with siRNA targeting CD25, enabling selective modulation of IL-2 signaling in CD8^+^ T cells and enhancing anti-tumor immunity [231]. Zheng et al. functionalized non-viral nanoparticles with aptamers targeting CD209 or immature dendritic cells (iDCs), achieving efficient *in vivo* delivery to circulating classical dendritic cells, a key population for initiating immune responses [232].

##### 4.2.2.5 Engineered proteins

Engineered protein binders, such as affibodies and DARPins (Designed Ankyrin Repeat Proteins), represent a promising class of small (∼6–15 kDa), stable, antibody-mimetic ligands that can be efficiently produced in bacteria with reduced cost and chemically conjugated to LNPs for targeted delivery [232]. Affibodies are engineered from the IgG-binding Z-domain of *Staphylococcus aureus* protein A. They are cysteine-free and exhibit rapid folding [233]. Idris et al. (non-peer-reviewed) developed CD5-targeting affibodies to deliver self-amplifying mRNA to T cells [234]. In a peer-reviewed study, anti-HER2 affibody-functionalized micelles were post-inserted into cisplatin-loaded liposomes, enhancing targeted cancer therapy efficacy [235]. DARPins are engineered protein scaffolds designed for specific target binding, combining small size, high stability, and efficient expression in bacterial systems [236]. Shipunova et al. showed that functionalization of liposomes with DARPins targeting HER-2 receptor achieved specific payload (barnase, a bacterial ribonuclease with cytotoxic activity) delivery to cancer cells [237]. Regarding immune cells, the field of DARPin targeting is growing with few publications or protected patents, showing for example efficient CD8^+^ T cells targeting [238].

In summary, immune cell-targeting moieties vary in size, manufacturability, and cost. Full-length antibodies (∼150 kDa) offer high specificity but require expensive production, may retain immunogenic properties and can easily saturate the LNP surface by their size [195,239]. Antibody fragments (Fab’, F(ab’)_2_, ScFv, VHH; 12–50 kDa) improve penetration, allow higher engraftment at the LNP surface while allowing easier conjugation, but can suffer from instability and residual immunogenicity [240,241]. Peptides (<5 kDa) are low-cost, tunable, and less immunogenic, though often cyclized to enhance stability and affinity [242]. Small molecules (<1 kDa), including carbohydrates and Siglec ligands, are scalable and minimally immunogenic, but require multivalency and are limited to specific cell types [243]. Protein scaffolds like affibodies (∼6.5 kDa) and DARPins (∼15 kDa) are stable, less-immunogenic, and easily produced in bacteria, yet remain underexplored for immune cells targeting, with most data confined to patents [244,245].

#### 4.2.3 Cell-specific expression via mRNA design

mRNA, by itself, in the absence of a cell-specific promoter, is always expressed wherever the cellular translational machinery is active. Even with the best shell optimization and state-of-the-art targeting ligands, 100% delivery to the intended target cell population remains unattainable. This limitation arises from off-target distribution of some proportion of the payload. Therefore, beyond optimizing delivery vehicles, it is often beneficial to incorporate an additional layer of specificity at the level of the mRNA itself (Fig. 6). Although most of the emerging strategies designed for such post-delivery regulation apart from miRNA-based approaches are still in early stages of development, particularly in immune cells or even within LNP systems, they hold strong potential to enhance specificity. This added layer of control is especially relevant for gene editing, where restricting expression to the desired cell type is essential to minimize/avoid off-target or deleterious effects.

##### 4.2.3.1 De-targeting through sequence-based strategies

Achieving tissue-specific mRNA expression remains a significant challenge, particularly due to the dominant hepatic uptake observed following intravenous administration of LNP. To overcome this, researchers have explored mRNA de-targeting strategies. Jain et al. introduced microRNA target sites (miRts) into therapeutic mRNAs to suppress translation in healthy tissues while preserving expression in diseased cells [246]. Specifically, they leveraged miR-122, highly expressed in healthy hepatocytes but downregulated in hepatocellular carcinoma. By doing so, they were able to deliver an mRNA encoding a toxic protein in hepatic cells, but allowed its translation only in diseased cells, miR-122 repressing translation in healthy liver tissue. Similarly, inserting miR-142 sites restricted expression in immune cells, such as macrophages. Expanding this concept, Brooke et al. proposed Multiorgan Protection (MOP) sequences consisting of UTR-embedded miRNA target sites, to confine mRNA vaccine expression to the site of injection [247]. Using a luciferase reporter system, this approach significantly reduced off-target expression in organs including liver, kidney, lung, pancreas and heart following both i.v. and i.m. administration. When applied to IL-12p70 mRNA vaccines, the strategy enhanced both humoral and cellular immunity while minimizing systemic exposure, thereby improving safety and promoting durable immunity in mice.

##### 4.2.3.2 Active translation through structure-based strategies

Structure-based mRNA targeting harnesses RNA secondary structures and programmable interactions to enable precise cell-type- or state-specific payload expression. The strategy is based on toehold switches embedded in the 5′ untranslated region (UTR) of the therapeutic mRNA. These switches form hairpin structures that block ribosomal access to the translation initiation site. They unfold only in the presence of a specific endogenous RNA, called the trigger RNA, expressed by the target cell. Binding between the toehold sequence and the trigger RNA initiates a strand displacement reaction that exposes the ribosome binding site, thereby activating translation. In parallel, riboswitches, either natural or engineered regulatory elements, regulate gene expression by undergoing conformational changes in response to small-molecule ligands. While toehold switches offer high programmability via RNA-RNA recognition, riboswitches provide an orthogonal mechanism for translation control through ligand-responsive folding, expanding the toolkit for post-transcriptional regulation in synthetic mRNA systems [248,249]. For instance, Lin et al. demonstrated the use of riboswitch by engineering guide RNAs where the binding of a small molecule (theophylline) to an aptamer triggered the removal of a blocking spacer sequence, allowing activation of sgRNA and enabling CRISPR-Cas9 gene editing in mammalian cells [250]. Siu et al. designed toehold-gated gRNAs (thgRNAs), where a stem-loop sequesters the gRNA spacer, keeping CRISPR-Cas9 inactive [251]. Upon cognate RNA trigger binding, branch displacement exposes the spacer, triggering Cas9 activation. This approach enables robust and orthogonal control using both synthetic or endogenous RNAs, including OxyS, RyhB, and full-length mCherry mRNA, without impairing the translation of the mRNA carrying the switch. Similarly, Hao et al. used toehold switches to reconfigure sgRNA structures, demonstrating reversible control of Cas9 activity *in vitro* [252]. Hashemabadi et al. developed Intelligent guide RNA (IngRNA), a multi-step guide RNA design that remains inactive until a specific RNA trigger is present. The crRNA is initially hidden by flanking sequences that block its structure and prevent target recognition. When the trigger RNA binds to the first toehold, it causes a structural change that exposes a second, previously hidden toehold. These stepwise unfolding releases the crRNA from its inactive state, allowing it to function. The use of two sequential toehold switches increases control and specificity, enabling conditional CRISPR activation based on cellular RNA inputs [253]. Zhao et al. expanded the toehold concept with eToeholds, resulting in synthetic RNA switches that enable conditional translation of a reporter gene in eukaryotic cells in response to specific trigger RNAs. These switches integrate engineered internal ribosome entry sites (IRESs) that remain inactive until base pairing with a cognate RNA trigger induces a structural change that permits translation. This approach allows robust and programmable sensing of endogenous RNAs, achieving high dynamic range and cell-type-specific gene expression in both mammalian cells and yeast [254].

In a distinct strategy, Rastfeld et al. developed selectively expressed RNAs (seRNAs), which are modular constructs that restrict translation in target cells [255]. In non-target cells, upstream ORFs (uORFs) and an IRES-blocking sequence (IB) inhibit translation. In target cells, a specific endogenous RNA marker induces partial seRNA degradation, removing the IB, allowing IRES refolding and translation initiation. This system also incorporates an RNase-inhibitory (RI) element for transcript stability. Using this design, the authors achieved a selective expression of active caspase-3 in keratin-positive breast cancer and glioblastoma cells, triggering apoptosis *in vitro* and *in vivo*, with no observed toxicity in healthy tissues.

##### 4.2.3.3 ADAR-based targeted translation

Another emerging strategy for achieving cell-specific expression exploits the activity of endogenous ADAR enzymes (Adenosine Deaminases Acting on RNA), which catalyze the deamination of adenosine (A) to inosine (I) within double-stranded RNA (dsRNA). Inosine is read as guanosine (G) during translation, enabling RNA-level recoding [256]. Building on this, Yang et al. developed CellREADR (Cell access through RNA sensing by Endogenous ADAR), a modular RNA sensing platform that enables selective expression of payloads in target cells [257]. The system consists of a single engineered transcript referred to as “readrRNA" containing a ∼250-nucleotides sensor domain complementary to a specific endogenous RNA, and along with an in-frame UAG STOP codon upstream of the effector coding sequence. Following hybridization with the target RNA, the resulting dsRNA structure recruits endogenous ADAR enzymes, which catalyze the conversion of a UAG stop codon to UGG, thereby restoring a tryptophan residue and enabling translation of the downstream protein. This design enables conditional translation based on the presence of specific cellular RNAs. In a related approach, Gao et al. introduced RADAR (RNA sensing using ADAR), which similarly harnesses endogenous ADAR activity to trigger therapeutic gene expression in response to defined RNA markers. By coupling the translation to the presence of defined cellular RNAs signature, these systems offer a powerful approach for highly selective and programmable targeting of diseased or distinct cell population [258].

Schematic outline approaches to improve immune cell specificity and translational regulation in LNP-mediated mRNA delivery. Surface modifications of LNPs, such as antibodies, nanobodies, peptides, small molecules, aptamers, and engineered scaffolds to enable selective immune cell targeting (left). These can function through direct receptor engagement, modulation of the protein corona, or alteration of lipid composition. Translational specificity is achieved at the mRNA level via cell-specific regulatory elements (right). These include miR-responsive sequences that induce degradation in off-target cells, as well as structure-based toehold switches or ADAR-mediated editing systems that enable translation selectively in target cells.

#### 4.2.4 Functionalization of NPs: short overview of conjugation strategies

Active targeting with targeted LNPs marks a key innovation in payloads release, enabling selective delivery to specific cells via receptor–ligand interactions. By incorporating recognition elements into the NP surface and combining them with optimized administration routes and advanced conjugation chemistries, this strategy enhances tissue specificity, efficacy, and safety in conditions previously intractable diseases [[259], [260], [261]]. Most of the developed targeted LNPs are based on a chemical linkage of a moiety to the nanoparticle. Among all the possible chemistries, the most commonly used are maleimide-thiol conjugation, NHS-amine coupling and SPAAC (Strain-Promoted Azide-Alkyne Cycloaddition) [262,263]. While commonly employed, the underlying rationale for choosing particular coupling chemistries is frequently underexplored. Inappropriate ligand attachment can result in ligand misorientation, reduced receptor affinity, premature detachment, increased immunogenicity, or compromised particle stability, potentially reducing therapeutic efficacy [264,265]. Addressing these considerations can guide rational design, improving the clinical potential of immune cell-targeted therapeutics.

##### 4.2.4.1 Maleimide-thiol conjugation

Maleimide–thiol conjugation, a type of Michael addition, is a widely adopted covalent coupling strategy used to functionalize LNPs particularly through PEG–lipids bearing maleimide groups and cysteine-containing ligands [266]. This reaction forms a stable thioether bond under mild, physiological conditions without the need for toxic catalysts. It allows precise control over ligand density, achieved by adjusting the molar percentage of PEG–maleimide in LNP formulations, while ensuring strong and durable covalent attachment. This strategy can be used for targeting many ligands and immune cells. For example, for antibody and fragments, Metzloff et al. demonstrated a dual conjugation of anti-CD3/CD28 thiol-containing antibody Fab' fragments to stimulate T cell activation for CAR-T cell production [267]. Tombácz et al. successfully targeted CD4-positive T cells with anti-CD4 antibodies enhancing targeted gene delivery [189]. Regarding peptides, Han et al. showed a 70-fold higher brain luciferase signal and 8 times increase in brain to liver ratio when they coupled brain-targeting peptides to LNPs *versus* non-targeted one [268]. However, a free thiol on the ligand is essential for maleimide-thiol conjugation, which may present challenges. Those are obtained either via cysteine residues or chemically using SATA, which adds a protected thiol to primary amines and requires deacetylation. This chemical route adds a synthesis step [269]. Moreover, unreacted maleimide can bind thiols on serum protein, impacting uptake [268], but also hydrolyze in aqueous media, reducing reactivity, while increasing immunogenicity, or instability [262,270].

##### 4.2.4.2 NHS-amine conjugation

N-Hydroxysuccinimide (NHS)-amine coupling is a well-established covalent bioconjugation method widely used in drug and gene delivery [271]. NHS esters react with primary amines under physiological conditions, forming stable amide bonds and releasing NHS as by product. Herrera-Berrera et al. used this method to couple LNPs to peptides targeting photoreceptors, enabling mRNA delivery to the retina of mice and non-human primates [272]. Li et al. used NHS-PEG-lipid with amine-containing mannose or chitosan to enhance mucosal vaccine delivery efficiency [273]. However, NHS coupling is non-specific, reacting with any free amine, and NHS esters hydrolyze in water within minutes to hours, requiring precise handling and purification. To address this, stable lipid-carboxylic acid (–COOH) can be used. This lipid exerts enhanced stability over NHS-lipid, allowing better storage. However, it requires activation with EDC/NHS to obtain NHS function at the LNP surface for further conjugation, adding handling steps and associated cost/quality controls [274]. The limitations of non-specific amine coupling have been highlighted in studies comparing it to more controlled strategies. Weller et al. showed that regioselective maleimide–thiol conjugation of half-antibodies onto nanoparticles allowed better antibody orientation, leading to stronger T cell activation at lower ligand densities compared to random NHS-based coupling. This demonstrates that the choice of conjugation chemistry can significantly influence the functional efficiency of antibody-functionalized delivery systems [275].

##### 4.2.4.3 SPAAC conjugation

SPAAC is a biorthogonal copper-free click chemistry reaction that enables the covalent coupling of molecules through the cycloaddition of azides and strained alkynes, most commonly cyclooctynes, such as DBCO (dibenzocyclooctyne**)** [276,277]. This reaction proceeds rapidly and selectively under physiological conditions and is particularly attractive for biomedical applications due to its biorthogonality, it does not interfere with native biomolecules. Tan et al. developed DBCO-LNPs to specifically bind azido moiety directly present at the plasma membrane of cancer cells, thanks to metabolic sugar labelling [278]. The same strategy can be employed to immune cells such as T cells as shown by Li et al. [279] Zhao et al. demonstrated that adding an DBCO-F4/80 antibody to Azido-LNPs allowed targeting of a specific surface marker of macrophages, both *in vitro* and *in vivo*, for successful delivery of mRNA and siRNA [194]. However, while SPAAC chemistry offers clear advantages in terms of selectivity and reaction kinetics, it also presents some notable drawbacks related to the hydrophobicity and bulkiness of DBCO which can interfere with LNP self-assembly, colloidal stability, and *in vivo* pharmacokinetics [277,278]. Therefore, azido-modified LNPs are mainly used to avoid aggregation or formulations issues. Moreover, as for maleimide-thiol conjugation, modification of ligands to make them suitable can be challenging.

##### 4.2.4.4 Impact of conjugation chemistry on nanoparticles behavior

Recently, the impact of these conjugation chemistries on LNP performance was assessed by the study of Jester et al., which directly compared various functionalized PEG-lipids to assess how surface chemistry alone shapes cellular interactions and biodistribution. Five LNP formulations were analyzed; one unmodified and four bearing PEG-lipids with different functional groups (folate, maleimide, carboxyl, PDP). By excluding conjugated ligands, the study focused on the intrinsic effects of functionalized lipids on physicochemical properties, cellular uptake, and tumor retention after i.t injection. Folate-modified LNPs rapidly associated with HepG2 cells via folate receptor binding but showed poor mRNA delivery. In contrast, maleimide LNPs exhibited delayed uptake yet improved transfection. Carboxyl and PDP groups conferred little cellular interaction or delivery activity. Increasing the proportion of functional lipids enhanced binding for folate and maleimide but reduced transfection, suggesting a trade-off between surface engagement and cytosolic delivery. *In vivo*, folate- and maleimide-modified LNPs showed greater retention at the tumor site, highlighting how surface reactivity and receptor interactions influence local biodistribution [280].

### 4.3 Translational bottlenecks of LNP-CRISPR delivery

The previous sections have outlined how compositional design, as well as surface engineering are progressively expanding the precision and reach of LNP-mediated CRISPR delivery. Yet several biological and formulation realities continue to separate engineered nanoparticles from clinical performance. These govern whether efficient *in vitro* transfection translates into durable, safe, and scalable *in vivo* gene editing. Four interconnected challenges deserve focused attention: the dynamic protein corona that redefines LNP identity upon entry into biological fluids; the persistently low efficiency of endosomal escape; the immunogenicity of both lipid shell and cargo; and the formulation constraints imposed by the increasing molecular size of next-generation editors.

#### 4.3.1 Protein corona: the moment LNPs leave the buffer

Despite the precision achievable through rational LNP design, a fundamental challenge remains: the particle that enters the body is not the same particle that was formulated. One of the most consequential transitions in LNP delivery occurs within seconds of contact with biological fluids. Particles formulated and characterized in defined buffers instantly acquire a heterogeneous layer of adsorbed biomolecules, the so-called biomolecular corona, which redefines their functional identity both *in vitro* and *in vivo*. This corona consists of a tightly bound “hard” corona composed of proteins with high affinity, surrounded by a more dynamic “soft” corona [281]. Together these layers can contain apolipoproteins, complement factors, immunoglobulins, vitronectin, fibronectin, and numerous other plasma constituents [282]. The composition of this corona, rather than the engineered lipid surface, governs how the particle is recognized by cellular receptors, where they accumulate and how efficiently their cargo is delivered. The canonical example of this principle is hepatic tropism: ApoE adsorption onto LNPs enables LDL receptor-mediated hepatocyte uptake, a mechanism underlying FDA-approved siRNA therapies but also the dominant cause of off-target accumulation when extrahepatic delivery is intended [[174], [175], [176]]. Recent study has further refined this model by showing that high-density lipoprotein (HDL) particles, rather than ApoE alone, serve as the primary source of functionally relevant corona ApoE. Multiomics analysis revealed that corona HDL content was a superior predictor of *in vivo* LNP activity than ApoE itself [283].

Importantly, specific corona proteins can uncouple cellular uptake from functional delivery. Vitronectin-enriched coronas increase surface association while reducing mRNA expression, whereas ApoE-enriched coronas drive substantial increases in cellular uptake yet simultaneously promote lysosomal co-localization, limiting net transfection [282]. This dissociation between internalization and productive delivery challenges the long-standing assumption that improved uptake necessarily translates into improved transfection. For immune cell-targeted formulations, this introduces an additional layer of unpredictability, as targeting ligands such as antibodies or peptides may be partially masked by absorbed proteins, redirecting particles toward off-target populations or weakening receptor engagement. Corona composition is further shaped by multiple formulation and delivery variables, including lipid identity (i.e. the SORT lipid, which dictates organ tropism through corona modulation) [184], PEG density and shedding kinetics, administration route, and the specific biological fluid encountered. Plasma-derived and cerebrospinal fluid-derived coronas yield markedly different cellular behaviors *in vitro* [284]. These observations suggest that corona formation should not be viewed as a passive post-formulation event, but as an active determinant of LNP biodistribution and potency.

A particularly underappreciated determinant is the patient's metabolic state. Tang et al. showed that elevated cholesterol levels, as observed in hypercholesterolemia, substantially reshape nanoparticle protein coronas by increasing the retention of apolipoproteins while reducing complement protein binding through altered protein-NP affinities [285]. This cholesterol-mediated corona enhanced hepatocyte uptake via SR-B1 and LDLR-driven internalization, but also elicited stronger inflammatory responses in macrophages compared to coronas formed under normocholesterolemic conditions. *In vivo*, hypercholesterolemic mice showed increased nanoparticle accumulation in the liver, spleen, and brain, together with reduced accumulation in the lungs, compared to healthy controls. These findings identify the metabolome, including disease-related small molecules, as an additional determinant of nanoparticle fate. This has direct implications for immune cell engineering. Patient physiological state, including metabolic comorbidities common in oncology and autoimmune disease populations, may influence LNP corona composition and thereby alter biodistribution, immune recognition, and delivery potency.

Such variables should therefore be considered in preclinical model selection, formulation screening, and translational assessment. Finally, species differences in plasma proteome composition further limit the direct extrapolation of murine corona data to human applications, reducing the predictive value of standard preclinical models.

Together, these findings argue that corona composition should be considered an integral parameter in the rational design of immune cell-targeted LNP platforms. This requires a shift from purely formulation-centric optimization toward a framework that accounts for the biological identity acquired by the particle *in vivo*.

#### 4.3.2 Endosomal escape

Even when LNPs successfully reach and enter the cell of interest, efficient expression of delivered mRNA remains far from guaranteed. Quantitative assays consistently place the escape efficiency below 10% for standard LNP formulations, with estimates ranging from 1 to 3% for internalized cargo reaching the cytosol to 3-7% depending on formulation and assay methodology used [[286], [287], [288]]. Because the cytosolic escape represents the dominant post-uptake bottleneck, even modest improvements produce disproportionately large gains in functional editing output [15]. The prevailing model attributes escape to protonation of ionizable lipids in the acidifying endosome, driving electrostatic interaction with anionic endosomal phospholipids and a phase transition toward an inverted hexagonal structure that destabilizes the membrane and permits cargo release [15,286]. Recent imaging work has substantially complicated this picture. Johansson et al. demonstrated that only a small fraction of RNA is released from damaged endosomes, that ionizable lipid and RNA frequently segregate during endosomal sorting, and that many galectin-marked endosomes contain no detectable RNA cargo, suggesting multiple distinct inefficiency steps rather than a single escapable barrier [289]. Productive escape occurs preferentially from early endocytic and recycling compartments, with cargo progressing into mature late endosomes largely non-productive in most cases [287]. Critically, efforts to enhance cytosolic escape are not without consequence: increased endosomal membrane disruption can trigger galectin recruitment and downstream cGAS-STING activation, posing an immunogenicity trade-off particularly relevant for immune cell engineering [286]. Notably, cell-type-specific determinants of endosomal escape efficiency in primary immune cells remain almost entirely uncharacterized, representing a critical knowledge gap given the central role of this step in determining functional editing outcomes.

A notable recent methodological advance is the LysoTag-based Lysosomal Barcoding approach of Jozić et al., [290] which enabled direct *in vivo* quantification of endosomal escape kinetics for the first time. Using this platform, the authors found that approximately 8% of an optimized branched ionizable phospholipid LNP reached the cytosol within 30 min of administration. This formulation outperformed the clinical benchmark LP01 eightfold for *in vivo* CRISPR-Cas9 editing of the TTR gene. Lysosomal proteomics from the same study identified Rab7-mediated late endosomal maturation as a negative regulator of escape, providing a potential mechanistic target for formulation-level intervention. The development of analogous quantitative tools for extrahepatic and immune cell compartments represents an important unmet need.

#### 4.3.3 Immunogenicity of LNPs

Beyond all the advances in LNP targeting for *in vivo* precision editing of immune cells, the problem of possible immunogenicity persists. Neither the lipid shell nor the RNA cargo is immunologically inert, posing a real challenge for therapeutic applications that require systemic or repeated dosing [291]. One of the most widely documented immune responses is the formation of anti-drug antibodies (ADAs) against PEG, which is routinely included as a PEG-lipid component to enhance circulation and colloidal stability [292]. These ADAs, especially anti-PEG IgM and IgG isotypes can rapidly bind PEGylated LNPs upon re-exposure, leading to the accelerated blood clearance (ABC) phenomenon and reduced therapeutic efficacy. Indeed, ABC-driven clearance reduces second-dose mRNA expression efficacy by 15–55% depending on PEG-lipid structure, with losses compounding across further administrations [160]. The consequences of repeated administration remain insufficiently understood and deserve closer investigation to support the safe use of LNPs across diverse contexts.

In some cases, anti-PEG IgE has also been implicated in allergic reactions and, even in some cases, anaphylaxis. Another mechanism is complement activation-related pseudoallergy (CARPA), a hypersensitivity-like reaction triggered by complement activation via IgM binding; these molecular processes can manifest as cardiopulmonary distress, hypotension, or even anaphylaxis in severe cases. Although rare, these reactions are a major safety concern, particularly in systemically administered LNP-based therapeutics. Importantly, a significant proportion of the general population already carries pre-existing anti-PEG antibodies, likely due to prior exposure to PEG-containing products, cosmetics, or pharmaceuticals [293,294]. These pre-existing antibodies may bind to LNPs at first and repeated administrations, reducing efficacy and raising the risk of immune-mediated adverse effects mentioned. Several strategies are in development to mitigate these responses. Cleavable PEG variants shed their corona upon endosomal acidification, directly limiting the antigenic surface available for anti-PEG IgM induction [295]. Zwitterionic polymers and pSar offer structural alternatives with reduced immunogenic footprints, as previously mentioned [296,297]. Importantly, antibody generation against LNPs is strongly regulated by both dose magnitude and administration route: i.v. and subcutaneous injections generate substantial dose-dependent anti-LNP antibody levels, whereas i.m. administration produces markedly lower and dose-independent responses, suggesting administration route itself is a tunable parameter for mitigating immunogenicity [298]. As the field moves toward possibility of repeated *in vivo* LNP-CRISPR dosing in immune cells, rational co-optimization of PEG-lipid structure, administration route, and dosing schedules will be as critical as delivery efficiency itself.

The integration of targeting moieties onto LNP-mRNA platforms is another important aspect for achieving cell- or tissue-specific delivery. However, these moieties, being exogenous, introduce additional layers of immunogenic complexity that must be meticulously evaluated and addressed to minimize ADAs [299]. Protein ligands (antibodies and fragments, affibodies, nanobodies®) are typically engineered for reduced immunogenicity, but their foreign origin and specific structural features (e.g., non-human sequences, linker peptides, linker PEG, or sequence similarity to bacterial proteins) can still lead to unwanted ADA responses. This highlights that “reduced immunogenicity" is not synonymous with “non-immunogenic" and necessitates rigorous de-risking strategies [[300], [301], [302]]. Aptamers and carbohydrates represent non-proteinaceous targeting ligands that generally exhibit lower intrinsic immunogenicity compared to protein-based moieties. However, the presence of soluble PRRs in the bloodstream, such as Mannan Binding Lectin (MBL), could also play a role in the distribution of mannosylated particles *in vivo,* [303,304] as it has been demonstrated *in vitro* that the presence of MBL in the transfection medium can inhibit the internalization and transfection of macrophages by mannosylated liposomes [305]. In the case of aptamers, some motifs could also be detected by PRRs, leading to potentially deleterious immune activation [306,307].

In addition to surface-level interactions, the cargo itself, particularly exogenous RNA, can also trigger unwanted immune activation through recognition by innate PRRs such as Toll-like receptors (TLR3, TLR7/8) and RIG-I-like receptors, which are particularly present in antigen-presenting cells like dendritic cells and macrophages [291]. This innate sensing results in secretion of inflammatory cytokines (e.g., IFN-α, IL-6) and upregulation of interferon-stimulated genes (ISGs), which can impair translation of the delivered mRNA and reduce therapeutic protein expression or editing efficiency. Such responses may be advantageous in vaccine settings due to their adjuvant-like effects, but for therapeutic gene editing, they represent a major barrier to tolerability, efficacy, and reproducibility. A widely adopted strategy involves addition of chemical modifications in mRNA, including incorporation of N1-methyl-pseudouridine, which has consistently demonstrated superior expression efficiency and reduced immunogenicity across multiple cell types [80,81,308]. There is also rigorous purification to remove double-stranded RNA (dsRNA) contaminants, which have been shown to significantly reduce innate immune recognition and improve protein expression profiles. Importantly, these findings were crucial for unprecedented success of mRNA vaccines. Nevertheless, it is important to have a context in mind as it was shown that in *ex vivo* engineering of the immune cells that unmodified mRNA can achieve equal or even higher transfection efficiency than modified mRNA in T and NK cells, likely due to their low expression of pattern recognition receptors and related adapter proteins [82,83]. These findings highlight the necessity of developing purpose specific formulations that balance transfection efficiency with biological effects and immunogenicity.

In summary, the immunogenicity of LNPs arises from both surface components (e.g., PEG-lipids) and the cargo itself (e.g., unmodified or impure RNA), posing a significant concern for systemic *in vivo* applications. While single low-dose i.v. administrations are generally well tolerated, higher doses or repeated injections may increase the risk of severe adverse events [309]. As previously noted, immune responses to Cas9 can also trigger the elimination of Cas9-expressing cells, further compromising therapeutic efficacy. By contrast, *ex vivo* LNP-mediated engineering avoids these immunological barriers, both at the level of the delivery vehicle and cargo (RNA and Cas9 protein) but introduces other bottlenecks and practical challenges. As the field aspires toward *in vivo* LNP-CRISPR dosing, mitigating ADA formation, CARPA, and innate immune sensing will be essential to ensure the safety, efficiency, and persistence of gene editing outcomes.

#### 4.3.4 Cargo complexity and formulation constraints in CRISPR-LNP delivery

A fourth, increasingly relevant constraint arises from the cargo itself rather than the carrier. Unlike conventional mRNA therapeutics encoding a single antigen or protein, CRISPR-based applications require coordinated intracellular delivery of multiple distinct molecular components. At minimum, this includes an editor-encoding mRNA and a guide RNA; template-dependent editing further needs a donor repair template. These components differ in size, structure, physicochemical properties, and functional requirements. This multi-cargo complexity introduces formulation challenges that scale non-linearly with the number of components and have no direct equivalent in single-cargo LNP systems [129,139].

The first constraint is cargo size. Cas9 mRNA at ∼4.5 kb is generally well-accommodated by standard LNP formulations. By contrast, base editor mRNA is larger, typically ∼5.0–6.2 kb, and prime editor mRNA is even larger, at ∼6.7–6.9 kb, representing a substantial increase in RNA length (35–53%) relative to Cas9 [126,310,311]. Whether this size increase meaningfully affects encapsulation efficiency and particle integrity remains poorly characterized, as systematic head-to-head comparisons across the Cas9, base editor, and prime editor mRNA size range are still lacking. This uncertainty is compounded by limitations in how encapsulation is routinely measured. Standard encapsulation efficiency percentage (EE%) calculations consistently exceed 85% regardless of cargo size, yet EE input% falls below 50% for benchmark formulations, meaning that RNA loss during formulation is systematically underreported [312]. As a result, the true formulation performance of larger editor mRNAs in current LNP platforms remains an open question. Nevertheless, large RNA encapsulation is feasible. Self-amplifying RNA of approximately 9500 nt has been successfully formulated in LNPs after dedicated optimization of lipid composition and formulation parameters, indicating that cargo size is a formulation constraint rather than an absolute barrier [313,314]. Parallel efforts in protein engineering are also beginning to reduce cargo burden: compact prime editor variants such as PE6a–g are 516–810 bp shorter than PEmax while maintaining, and in some settings improving, editing efficiency in primary human T cells [310].

A second constraint is stoichiometric co-encapsulation. Productive editing requires that the editor mRNA and guide RNA reach the same cell, ideally within the same particle or within a particle population that supports efficient co-delivery. Single-particle analysis has revealed that even under optimized conditions, only 50–60% of LNPs co-encapsulate both RNA species, with 30–36% carrying guide RNA alone and contributing nothing to editing [315]. Conventional bulk metrics including particle size, PDI, and total encapsulation efficiency fail to capture this heterogeneity. In one study, three mixing methods produced LNPs with near-identical biophysical profiles, yet produced markedly different *in vivo* editing efficiencies, with indel rates of 36.3% versus 55.4%. This difference was driven solely by variation in RNA copy numbers per co-encapsulated particle rather than by standard formulation parameters [315]. Notably, the optimal balance between cargo concentration per particle and population homogeneity is not universal and requires specific optimization for each combination of editor mRNA and guide RNA or pegRNA, as well as the intended application and dosing regimen. These findings argue that single-particle payload distribution should be adopted as a critical quality attribute for CRISPR-LNP systems, alongside conventional bulk analytics.

A third constraint arises specifically in HDR-based approaches, where an exogenous donor template must be co-delivered alongside the nuclease machinery. Unlike single-stranded RNA, dsDNA donor templates adopt a rigid double-helical structure that is poorly matched to the packing geometry of standard ionizable LNP optimized for flexible nucleic acids [316]. This structural mismatch leads to non-standard particle morphologies and necessitates formulation re-optimization beyond what RNA-only systems do. Encapsulation of large dsDNA has been shown to produce distinct multilamellar architectures with alternating lipid bilayers rather than the canonical core-shell structure. The therapeutic size requirements for HDR templates further compound this challenge. Homology arms of 200–500 bp flanking the insert of interest are required for efficient recombination, and for clinically relevant payloads such as CAR constructs of ∼1.6 kb, total donor template size can reach several kilobases, placing it near the upper practical range of what any LNP platform can currently accommodate alongside RNA cargos [317]. Short ssODN templates circumvent some of these encapsulation constraints by their smaller size and reduced rigidity. However, their utility is inherently limited to small sequence corrections and cannot support large knock-in strategies. Nanoplasmids and minicircular DNA reduce bacterial backbone content compared to conventional plasmids, improving cell viability and editing performance in primary T cells, but they do not eliminate the intrinsic size burden imposed by the therapeutic insert and homology arm [318]. Delivered dsDNA also introduces an additional immunological liability, as cytosolic DNA can activate the cGAS–STING pathway, a constraint not shared by RNA cargos and one that may further limit tolerability and editing yield in primary immune cells [319]. As proof of concept, Foley et al. achieved 3–3.5% site-specific CFTR integration in airway epithelial cells using LNP co-delivery of Cas9 mRNA, sgRNA, and a full-length linear dsDNA donor, with nuclear trafficking identified as the primary remaining bottleneck [320]. Whether similar approaches can be adapted to immune cell targets remains uncertain, particularly because nuclear access and HDR activity are typically more restrictive in these cells than in dividing epithelial models.

RNP delivery introduces yet another layer of formulation complexity. Standard LNP formulation conditions including acidic aqueous buffers and organic solvents during microfluidic mixing can compromise protein integrity. RNP-LNP formulation therefore often requires neutral or mildly acidic buffer systems, inclusion of cationic lipids to facilitate RNP-lipid interaction, and in some cases engineering of thermostable Cas9 variants that better withstand encapsulation conditions [[321], [322], [323]]. In a direct comparison, Walther et al., [322] found that mRNA-LNPs outperformed RNP-LNPs in both settings: *in vitro,* mRNA-LNP achieved higher editing efficiencies across cell lines with earlier onset of gene correction. *In vivo*, they yielded 60% knockout in hepatocytes, whereas all animals treated with RNP-LNP died within 20 h post-injection due to particle aggregation at relevant RNP concentrations. Partial surface association of the RNP rather than core encapsulation was identified as the likely driver of this instability, representing a critical unresolved constraint for RNP-LNP co-delivery of HDR templates.

Altogether, these constraints are interdependent and can amplify one another. Critically, each is amplified in immune cell targets relative to hepatocytes – the cell type against which most LNP platforms are benchmarked and optimized. Larger editor transcripts, multi-component co-encapsulation, donor template rigidity, RNP instability, and immune sensing each impose distinct formulation pressures, but their combined effects are only beginning to be systematically characterized. These challenges are especially relevant for immune cell targets, where delivery, nuclear access, innate immune activation, and editing-associated fitness costs are generally less favorable than in hepatocytes, the benchmark cell type for most LNP platforms. Addressing this gap will require coordinated optimization of formulation chemistry, cargo format, analytical characterization, and dosing strategy in the context of the intended target cell biology.

## 5 Immune cell targets and therapeutic applications

Immunotherapy has transformed modern medicine by leveraging the immune system to combat cancer, autoimmune diseases, and chronic infections. Among these, cancer immunotherapy has made particularly striking advances, with broad range of modalities, from small molecules and cytokines to checkpoint inhibitors, monoclonal antibodies, vaccines, and adoptive cell therapies [324]. Even though immune-checkpoint inhibitors have produced notable tumor regression results in clinics, resistance to such therapies is frequent due to compromised and scarce immune cells, as well as suppressive tumor microenvironment, poor infiltration, and tumor heterogeneity [325]. In contrast, adoptive cell transfer (ACT), which includes (re-)introducing engineered immune cells, such as CAR-T cells, into the patient, can address those concerns and has shown great success in treating hematologic malignancies, with its applications in solid tumors treatment still in its early stages [326]. Traditionally, ACT relies on *ex vivo* modification and expansion of patient-derived cells, which can be time-consuming and costly. Indeed, *ex vivo* immune cell handling and activation are a critical determinant of the success of genetic engineering, as it enhances metabolic activity, upregulates receptors relevant for vector uptake (for instance Low-Density Lipoprotein Receptor (LDL-R), ApoE receptor relevant to uptake of LNPs), and promotes translation of delivered mRNA [[327], [328], [329]]. However, this artificial activation can also disrupt essential cellular processes, such as autophagy, as observed in CAR-T and NK cells. Impaired autophagy has been linked to metabolic dysfunction, reduced cell persistence, and diminished anti-tumor efficacy; effects that can be mitigated by restoring autophagy [330]. Therefore, because the full complexity of native immune activation *in vivo* cannot be reproduced *ex vivo*, artificial stimulation risks redirecting cell fate and impairing functional responsiveness. To overcome these limitations, emerging *in vivo* gene delivery approaches aim to bypass these limitations by directly programming immune cells inside the body, potentially streamlining manufacturing and broadening accessibility [331]. CRISPR-Cas genome editing systems have become central to this evolution [1].

**Fig. 7:**
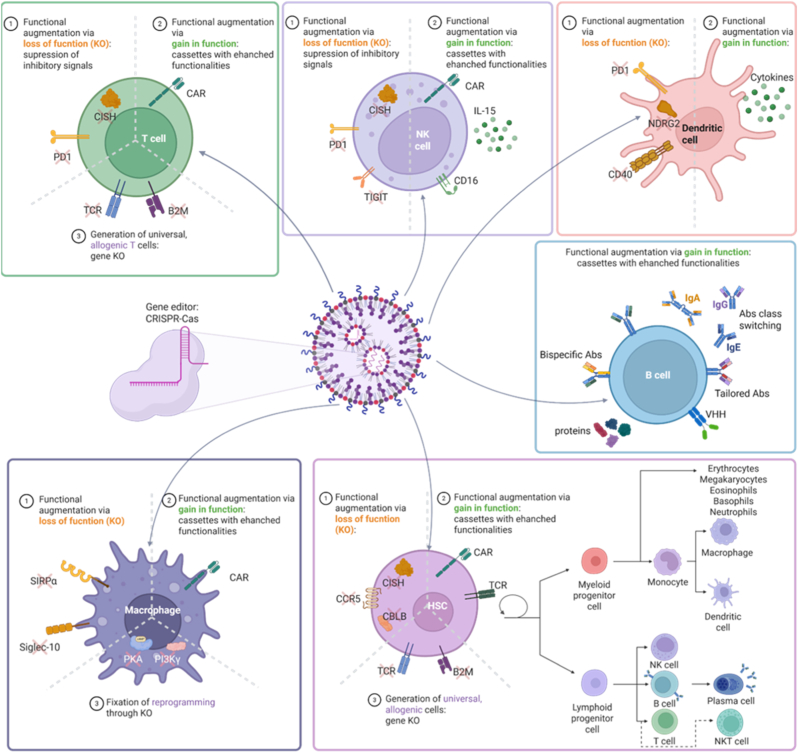
CRISPR-based engineering strategies across immune cell types.

In the sections that follow, we will explore each major immune cell type by: (i) summarizing its core immunological roles and relevance in immunotherapy; (ii) highlighting gene-editing strategies that enhance or rewire its function; (iii) reviewing nanoparticle-based delivery systems, with a particular focus on LNPs, in terms of transfection efficiency, scalability, immunogenicity, and translational potential; and (iv) examining current advances in nanoparticle-mediated gene editing, focusing on how these platforms are being leveraged to reprogram immune cells for therapeutic applications (Fig. 7).

### 5.1 T cells

T lymphocytes orchestrate adaptive immunity, with CD8^+^ cytotoxic T cells eliminating infected or cancerous cells, as well as CD4^+^ helper and regulatory T cells (Tregs) modulating immune responses. Their central role in immune surveillance and memory response makes T cells a cornerstone of cancer immunotherapy, infectious disease control, and autoimmunity. This is highlighted by the FDA approval of six CAR T cell therapies for B cell malignancies and a growing number of clinical trials investigating their use in autoimmune diseases and solid tumors [35,332,333]. Chimeric antigen receptors (CARs) are synthetic constructs comprising an antigen-binding domain, transmembrane segment, and intracellular signaling module, designed to reprogram various immune cell types to recognize and eliminate cells expressing specific surface antigens, such as CD19 in malignant B cell populations.

CRISPR technology has emerged as an indispensable tool to reprogram T cells for therapy, with several clinical trials already underway as mentioned earlier. It is used to enhance T cell function, either by deleting the checkpoint receptors (PD-1) or by targeting intracellular immune checkpoints/mediators like *CISH*, which has entered clinical trials for improving T cell function in solid tumors (NCT03538613), but also by inserting either a cancer-specific T cell receptor (TCR) or CAR construct into the endogenous TCR locus [3,25,26]. Likewise, CRISPR gene editing has enabled the generation of “universal” T cells by simultaneously disrupting the endogenous TCR and HLA genes, minimizing graft-versus-host disease and immune rejection, key advances in the development of allogeneic, “off-the-shelf” CAR T cell therapies [42,46,334]. These are just some of the examples illustrating how CRISPR platforms are being harnessed to endow T cells with superior therapeutic traits.

In most cases, CRISPR components are delivered via EP of RNPs or viral vectors [35,96]. However, emerging evidence highlights the strong potential of lipid LNPs and polymer-based systems for T cell transfection. One early demonstration involved Charge-Altering Releasable Transporters (CARTs), which efficiently delivered cargo to primary T cells *ex vivo* and enabled selective *in vivo* transfection with 97% spleen tropism, without requiring targeting ligands [335]. In a direct comparison with EP, LNPs enabled superior *ex vivo* delivery of CAR-encoding mRNA into primary T cells, resulting in extended CAR expression, reduced exhaustion, and prolonged functionality [115]. This enhanced performance is attributed to a less cytotoxic, receptor-mediated uptake mechanism and gradual mRNA release, establishing LNPs as promising vector for T cell engineering. Mitchell's group has systematically advanced LNP technologies for *ex vivo* mRNA delivery to T cells, identifying C14-4 as a top-performing ionizable lipid capable of outperforming lipofectamine and EP in both luciferase and CAR mRNA delivery, with lower cytotoxicity [145]. Through design-of-experiment (DoE) approach, they optimized the molar ratios of C14-4-based LNPs, yielding the B10 formulation, which achieved over 80% CAR expression in primary human T cells and potent leukemia cell killing, comparable to electroporated and lentiviral CAR T cells [144]. Building on this, they engineered activating LNPs (aLNPs) by conjugating CD3/CD28 antibody fragments to the LNP surface, enabling one-step activation and transfection of T cells with ∼84% CAR^+^ cells, robust proliferation, and effective tumor clearance in a leukemia xenograft model [267]. Similarly, Papp et al. developed CD47/tLNPs by decorating LNPs with a CD47-derived “don't eat me” peptide and anti-CD4 antibodies, enabling *in vivo* delivery to CD4^+^ T cells, while evading hepatic clearance via suppression of Kupffer cell phagocytosis [217]. This strategy enhanced T cell transfection efficiency to over 51% in the spleen while simultaneously reducing liver uptake by 3-fold, setting a new benchmark for extrahepatic mRNA delivery in systemic applications. Together, all these studies (and many more reviewed in work by Pint et al. [336]) validate NPs, especially LNPs as clinically scalable and modular delivery systems for both *ex vivo* and *in vivo* T cell engineering and provide a strong foundation for adapting these platforms to deliver gene-editing cargos.

Building on these advances, Vavassori et al. demonstrated LNPs enable efficient and less toxic *ex vivo* CRISPR-Cas9 editing of human primary T cells compared to EP [116]. Using the commercial LNP formulation GenVoy-ILM T Cell Kit, the authors delivered Cas9 mRNA and sgRNA targeting *CD4*0LG (targeted for HDR-based correction in immunodeficiencies) and B2M, achieving up to ∼75% indels, comparable to EP. Critically, LNP delivery preserved cell viability with minimal apoptosis/necrosis, mitochondrial integrity, and supported early T cell expansion, doubling the yield of edited cells at 24 h post-treatment. The T cells retained memory-like phenotypes and IFN-γ response without signs of exhaustion. The study employed a combinatorial approach by pairing LNPs with AAV6 vectors carrying the HDR template. Although HDR efficiency was modest compared to EP, the higher early yield of viable, edited cells compensated for this limitation. Geczy et al. reported LNP platform for CRISPR-Cas9 delivery to primary human T cells, achieving up to 90% double KO of TCR and CD52 with >90% viability. This approach targets key immunogenicity markers, aligning with universal CAR-T strategies, and was successfully scaled from small culture formats to G-Rex bioreactors, supporting clinical translation [337]. In another approach, liposomes were employed for effective PD-1 KO in T cells [338]. More recently, Cevaal et al. introduced a LNP X formulation (SM-102/β-sitosterol) that enables efficient mRNA delivery to resting CD4^+^ T cells [339]. Using this platform, the authors first demonstrated efficient CRISPR activation (CRISPRa) delivery, inducing CD25 expression in up to 25% of primary CD4^+^ T cells and selectively reactivating latent HIV, with up to a 112-fold increase in spliced HIV transcripts without inducing T cell activation or toxicity. Although not yet tested *in vivo*, this non-invasive, DSB-free approach enables precise reprogramming of quiescent T cells and offers a promising foundation for targeted *in vivo* gene modulation using LNPs.

These goals are being directly addressed by Tessera Therapeutics, which is advancing a transformative gene editing strategy based on targeted T cell-tropic LNPs designed to deliver their proprietary Gene Writers. As already mentioned, at the core of this technology is target-primed reverse transcription (TPRT), that uses an RNA-encoded reverse transcriptase/endonuclease to nick genomic DNA and reverse transcribe a co-delivered RNA template directly into the DNA at the target site. Unlike traditional CRISPR systems that rely on DSBs, TPRT enables the precise insertion and editing of genetic templates (from transgene insertions to single nucleotide changes) without inducing DSBs, offering greater safety, control, and the ability to rewrite substantial segments of DNA [63,64,340]. Initial tests of T cell-targeted LNPs carrying a Gene Writer and a CAR template achieved an average of 16% CAR^+^ T cells in pre-activated human T cells, which exhibited potent cytotoxic activity and expansion *ex vivo*. Remarkably, in non-activated (resting) T cells, *in vitro* delivery of CD19 and CD20 CAR templates yielded up to 40% and 60% CAR^+^ cells, respectively, showing strong functional responses and successful editing in both human and non-human primate cells. *In vivo*, a single infusion of targeted LNPs carrying Gene Writers produced 30% CAR-T cells in xenograft mouse models, leading to effective tumor clearance and B cell depletion, and up to 30% B2M KO, highlighting the platform's therapeutic potential (as claimed in their latest press release from May 2026).

These advances highlight the feasibility of LNP-mediated T cell engineering, both *ex vivo* and *in vivo*. However, realizing their full clinical potential will require precise control over targeting, editing fidelity, and long-term T cell persistence and function. The coming years will determine whether these platforms can match the safety and reliability standards demanded by next-generation immunotherapies.

### 5.2 NK cells

NK cells are cytotoxic lymphocytes (defined as CD56^+^ CD3^−^) of the innate immune system, that eliminate virally infected cells and tumor cells without prior sensitization. In addition to their direct killing ability, they also produce key cytokines such as IFN-γ and TNF-α, which modulate immune responses and enhance antigen presentation. NK cells detect abnormal cells through downregulation of MHC I, typically present on healthy cells, or upregulation of stress-induced ligands, and can mediate antibody-dependent cellular cytotoxicity (ADCC) via CD16. Overall, their activation is governed by a dynamic balance between signals from activating and inhibitory receptors [341].

NK cells are increasingly explored for cancer immunotherapy due to their ability to target tumor cells, including those that evade T cells via MHC downregulation. NK cell-based therapies, including NK cell infusions and CAR-NK cells have shown safety and efficacy in treating malignancies, with reduced risk of GvHD and cytokine release syndrome (CRS) compared to T cells. In a recent phase 1/2 clinical trial (NCT03056339) on 37 patients with CD19^+^ B cell malignancies, allogenic cord blood-derived CAR/interleukin-15 (IL-15) NK cells achieved clinical responses comparable to those of CAR-T cell therapy, but without any cases of severe cytokine release syndrome, neurotoxicity, or GvHD [342]. One of the biggest advantages of NK cell therapies is their scalability: they can be developed from irradiated NK-92 cell lines or allogeneic primary NK cells derived from various sources, enabling the production of “off-the-shelf” therapeutic products that simplify manufacturing and reduce costs. Notably, allogeneic NK cells may be more effective than autologous NK therapies, avoiding functional exhaustion in immunocompromised patients [343,344]. Beyond CAR engineering, strategies such as IL-2 or IL-15 gene delivery aim to enhance NK cell persistence and activity in the tumor microenvironment [345].

Gene editing further enhances NK cell function by targeting inhibitory pathways and boosting metabolic fitness. For instance, CISH KO removes a key negative regulator of IL-15 and IL-2 signaling, leading to enhanced metabolic fitness, proliferation, and anti-tumor activity in NK cells, while also limiting their exhaustion [346,347]. Daher et al. showed that dual strategy of CISH-KO in IL-15–secreting CAR-NK cells improves metabolic fitness permitting greater *in vivo* persistence and cytotoxic function [348]. To go even further, CRISPR approach can enable KI of CAR or activating cytokine at the CISH locus, resulting in both CISH KO and additional functional activation at a time when the cell would normally suppress activity. Recent work by Wang et al. showcased multiplex base editing using up to six sgRNAs, with optimal tumor killing after simultaneous KO of TIGIT, PD-1, and CISH. They also demonstrated gain-of-function mutations by using BEs in form of introducing non-cleavable CD16a which improved ADCC-mediated killing and cytokine production in NK cells [41]. In a similar approach, PD-1-KO NK cells showed notably enhanced cytotoxicity and cytokine secretion *in vitro* and *in vivo*, decreasing tumor burden [349]. SOCS3-KO in NK cells showed higher cell proliferation and enhanced anti-tumor activity [350].

In the same manner as in T cells, gene delivery to NK cells, particularly for CRISPR-mediated KO/KI, still largely depends on EP and viral vectors, which show variable efficiencies [351]. For instance, efficient gene KI was achieved using rAAV6 as a template for HR (both of reporter genes as well as CD33-targeting CAR cassette) combined with EP of Cas9 mRNA/RNP - sgRNA [349,352]. A newer approach introduced by Xie et al. involves the GATALYST platform, which utilizes circular single-stranded DNA (cssDNA) template, enabling high-efficiency, non-viral gene KI in primary NK and T cells. When co-electroporated with CRISPR-Cas RNPs (Cas9, Cas9 nickase, and Cas12a), this platform supported the generation of functional CAR-T and CAR-NK cells with KI efficiencies exceeding those achieved with traditional dsDNA templates, while also markedly reducing cytotoxicity and inflammatory responses, often associated with dsDNA templates [353].

Despite the success of NP-mediated delivery in other immune cell types, NK cells have remained notably difficult to transfect. Delivery to NK cells is hindered by their limited endocytic uptake, low efficiency of endosomal escape due to neutral endosomal pH, and the risk of triggering innate immune responses via PRRs [343]. CARTs achieved mRNA transfection efficiencies of 20–40% in resting primary NK cells, with higher viability (between 60% and 85%) and minimal disruption of NK cell phenotype and function compared to EP. [114]. More recently, lipid-based nanoparticles have shown promise for mRNA delivery to NK cells. Nakamura et al. extended their siRNA delivery platform by developing CL1H6-LNPs, which demonstrated high gene silencing activity and achieved up to 100% EGFP expression in NK-92 cells [354,355]. Similarly, Douka et al. showed that LNPs formulated with SM-102 and Lipid5 achieved nearly 100% EGFP expression in NK-92, KHYG-1, and primary NK cells, outperforming EP [356]. Building upon these findings, Golubovskaya et al. reported the generation of approximately 75% CAR-expressing primary NK cells, following a protocol involving activation, expansion, and transfection using SM-102-based LNPs [357]. In our own work, we employed imidazole-based cationic liposomes and LNPs, achieving transfection efficiencies comparable to or exceeding those of SM102-based systems. Delivery of IL-2 mRNA using these platforms extended NK cell viability while preserving activation markers and cytotoxic function in both NK cell lines and primary NK cells [83].

These platforms also supported Cas9 mRNA and sgRNA delivery for gene KO. Nguyen et al. further enhanced KI efficiency using polyglutamic acid (PGA) to stabilize Cas9 RNPs and truncated Cas9 target sequence (tCTS)-modified HDR templates, improving editing across NK cells, γδ T cells, B cells, and CD34^+^ HSPCs [358]. This demonstrated that nanoparticle-based formulation of CRISPR components can significantly improve genome editing outcomes across diverse and clinically relevant immune cell types.

Whereas *in vivo* targeted delivery to T cells using LNPs has been extensively explored, analogous strategies for NK cells remain comparatively underdeveloped. Wu et al. recently reported that cholesterol-free LNPs formulated with carbonate-bearing ionizable lipids enabled 21% NK cell transfection *in vivo* in the spleen, outperforming previous LNPs [359]. However, specificity was lacking, with transfection observed across immune cell types. Targeting NK-specific markers (e.g., NKp46, CD56) offers a potential solution but poses challenges, as it may trigger unwanted activation or apoptosis. In contrast, targeting non-activating receptors (e.g., ASCT1/2) may provide safer and more controlled route for NK-directed delivery [343]. To date, no published study has demonstrated efficient *in vivo* CRISPR editing specifically of NK cells. However, NK cells may undergo incidental or bystander editing *in vivo*, particularly when LNPs are designed to target hematopoietic stem cells (HSCs), T cells, or broader anatomical sites such as the spleen.

Taken together, *ex vivo* LNP-mediated editing remains the most practical approach for NK cell engineering. This strategy involves isolating NK cells, treating them with LNPs loaded with CRISPR-Cas9 components or mRNA, potentially in multiple rounds during expansion, and subsequently infusing the edited NK cell product, which can also be administered as an allogeneic, “off-the-shelf” therapy. In contrast, *in vivo* LNP delivery to NK cells remains an aspirational goal, contingent upon further advancements in cell-specific targeting technologies.

### 5.3 B cells

B cells are central architects of humoral immunity. They produce antibodies, present antigens and secrete cytokines that shape both T cell responses and innate immunity. Upon encountering antigen and getting CD40L co-stimulation, they can undergo B-cell specific process including somatic hypermutations (SHM) and antibody class switch recombination (CSR). These processes lead to the production of high-affinity antibodies with specific effector function. Their differentiated progeny, antibody-secreting plasma cells, can survive for decades and continuously manufacture grams of protein per day, while memory B cells can be recalled repeatedly to renew this output upon re-exposure to antigen [360]. These “protein factories” can, in principle, be re-tasked to deliver therapeutic payloads, ranging from antibodies which are one of the most established (immuno)therapeutic interventions to enzymes, at steady-state levels that surpass conventional infusion strategies [361,362].

The approval of therapies that target B cells such as anti-CD20 antibody used for lymphoma and autoimmune disease, underscores that modulating B cells can have powerful clinical effects [363]. Gene editing now offers a more precise and versatile approach: not only depleting B cells but also reprogramming them to acquire novel therapeutic or protective functions. For instance, Immusoft and Be Biopharma exploit B cell engineering prior to plasma cell differentiation to produce therapeutic proteins for treating Mucopolysaccharidosis type I and Hemophilia, respectively. Both approaches are currently being evaluated in clinical trials (NCT05682144, NCT06611436) [362].

A central focus in the field is the generation of engineered B cells capable of producing designer antibodies for the treatment of cancer and infectious diseases. This can be achieved by integrating antibody cassettes into safe-harbor loci or by directly modifying the immunoglobulin (Ig) locus. In a proof-of-concept study, Hill et al. used electroporation of Cas9 RNPs followed by AAV6 donor delivery to insert CD19×CD3 or CD33×CD3 bispecific antibody cassettes into the CCR5 locus, achieving approximately 10% KI efficiency [364]. The resulting plasma cells continuously secreted bispecific antibody *in vivo*, and a single infusion led to complete leukemia remission in mice by activating T cell-mediated killing, similar to CAR T cells. In a parallel workflow, α-PD-1 IgG cassettes were integrated into the GAPDH locus of primary B cells with approximately 20% efficiency using BaEVTR-pseudotyped integrase-defective lentiviral vectors (IDLVs). The engineered plasma cells populated the bone marrow niche and continuously supplied the checkpoint inhibitor, restoring effector T cell function and delaying melanoma growth in murine model [365].

Another key area of focus is the CRISPR-based engineering of B cells to express pathogen-targeting antibodies for infectious diseases such as respiratory syncytial virus (RSV), influenza, and particularly human immunodeficiency virus (HIV; e.g., 3BNC117) [[366], [367], [368]]. These strategies use HDR to insert antibody cassettes into the IgH locus, typically via RNP and AAV6, achieving 10-30% KI efficiency *ex vivo*. After adoptive transfer and antigen boost, engineered B cells expand *in vivo* and undergo CSR and SHM, yielding affinity-matured plasma cells and long-living memory B cells. In HIV models, this enabled sustained neutralizing antibody production with continued maturation, while in RSV models, engineered B cells conferred near-complete protection in challenged immunodeficient mice. Unlike prior strategies that insert full-length heavy and light chains in the IgH locus, Rogers et al. generated functional single-chain antibodies with modular antigen recognition domains (such as VHH, scFv, or CD4 domains for HIV targeting) to minimize risks of autoreactivity due to light chain mispairing [369]. Nahmad et al. demonstrated that mature B cells can be directly reprogrammed *in vivo* to secrete anti-HIV antibodies through dual-AAV-mediated CRISPR KI at the IgH locus. This enabled durable, antigen-responsive humoral immunity with SHM and CSR [370]. AAV biodistribution studies revealed retention in non-lymphoid tissues, such as the liver and blood, which was mitigated by using a B cell specific CD19 promoter to restrict Cas9 expression and minimize off-target activity. In contrast to direct B cell editing, Castelli et al. demonstrated that *ex vivo* non-viral Cas12a KI into non-human primates (NHP) HSCs enabled engraftment, B cell differentiation, and durable anti-HIV antibody production in mouse model [371].

To date, several nanoparticle-based strategies have been explored to enable B cell engineering. For instance, Keim et al. reported the use of polycationic transfection agents called “nano-stars”, which achieved plasmid DNA transfection of up to 40% of primary B cells, while maintaining 70% of cell viability. Still, this method requires 370-fold less DNA to reach transfection efficiencies comparable to those obtained with electroporation [372]. In another study, poly-L-glutamic acid-based NPs have been used to stabilize and deliver RNP to multiple immune cells, B cells included [358]. Li et al. developed NPs based on PEG-PLGA block copolymer that show good uptake across multiple mature B cell subsets in spleen, lymph nodes, and bone marrow [373]. These NPs enabled *in vivo* delivery of CRISPR-Cas9 plasmids, achieving up to 30% indel efficiency at the *B220* locus. Functional knockout of BAFFR resulted in significant B cell depletion and therapeutic benefit in a murine model of rheumatoid arthritis. However, the study lacked comprehensive biodistribution analysis beyond lymphoid tissues and did not directly confirm BAFFR editing at the molecular level, instead relying on indirect phenotypic readouts.

When it comes to LNPs, it has already been shown that naked, spleen-tropic LNPs based on the OF-Deg-Lin ionizable lipid have been shown to drive significant protein expression in B lymphocytes following *in vivo* administration [374]. In a recent study, Suzuki et al. developed LNPs enriched with 15 mol% DSPC, enabling efficient and selective mRNA delivery to splenic B cells while avoiding hepatic transfection. Unlike many LNPs that depend on ApoE, this formulation relied on complement proteins, particularly C3, for B cell targeting via CD21/35 receptor-mediated uptake [375]. A recent patent publication (WO2025085695A1) demonstrated successful transfection of primary B cells using MC3-based LNPs, with EGFP expression in 20 – 65% of cells [376]. This highlights both the feasibility of LNP-mediated delivery to B cells and the fact that this area remains largely underexplored, offering significant potential for further development.

Despite these advances, LNP-based gene editing of B cells remains essentially unexplored. Systematic benchmarking of LNP platforms against electroporation and viral vectors in functional B cell readouts represents a near-term priority for the field.

### 5.4 Hematopoietic stem cells

Although not classified as immune cells *per se*, hematopoietic stem (progenitor) cells (HSPCs) are the self-renewing root of the entire hematopoietic system, capable of lifelong self-renewal and differentiation into all myeloid and lymphoid lineages; ultimately giving rise to the full spectrum of immune cells [377]. Phenotypically, the most primitive human HSCs are long-term HSCs (LT-HSCs), and they reside in the bone-marrow niche as CD34^+^ CD38^−^ CD90^+^ cells. Loss of CD90 or acquisition of CD38 marks transition to short-term HSCs and multipotent progenitors that will eventually give rise to T cells, NK cells, B cells (lymphoid lineage) as well as dendritic cells and monocytes (myeloid lineage) [378].

Critically, harvesting a patient's own CD34^+^ HSCs, engineering them *ex vivo*, and reinfusing them after conditioning enables durable, multilineage hematopoietic reconstitution. This autologous approach avoids the need for donor matching and mitigates the risks of GvHD [379]. This is the basis of gene and cell therapy approaches that were in the first place used to treat conditions like sickle cell disease, β-thalassemia, and immunodeficiencies. CRISPR editing has been essential for unlocking the full therapeutic potential of HSCs. The first approved CRISPR-based therapy for HSCs, intended for severe hemoglobinopathies, is exagamglogene autotemcel (Casgevy). This therapy involves *ex vivo* EP of CRISPR-Cas9 into the patient's CD34^+^ HSCs to cut the BCL11A erythroid enhancer re-activating fetal hemoglobin (HbF, α_2_γ_2_). The resulting HbF compensates for defective adult hemoglobin, effectively neutralizing disease symptoms and eliminating transfusion dependence for severe sickle-cell and β-thalassemia patients [7,380]. New therapies such as EDIT-301 (AsCas12a KO of the HBG promoter) and BEAM-101 (base editing that activates HBG) aims to induce HbF without creating DSBs [381,382]. This is important as each DSB activates the MRN-ATM-p53 axis which can drive p21-mediated apoptosis or senescence in LT-HSCs [69,383]. To limit this risk, gene editors should be delivered transiently and restricted to single-strand nicks or base substitutions (BEs and PEs), to avoid p53 activation and preserve stem-cell fitness.

Beyond haemoglobinopathies, HSC editing can seed the immune system with designer effector cells. This is particularly valuable for lineages that are naturally scarce, such as invariant natural-killer-T (iNKT) cells. For instance, Li et al. engineered CD34^+^ HSCs with an iNKT TCR and CRISPR-KO of B2M and *CIITA*, creating HLA-null “universal” HSC-iNKT cells that are both allogenic and fully resistant to host T cell-mediated allorejection [384]. HSCs can also be programmed to express CARs, leading to multilineage CAR progeny (e.g., CAR-T, CAR-NK, CAR-macrophages, CAR-neutrophils) for combinatorial targeted immunotherapy [385]. For instance, Carrillo et al. demonstrated that HSCs engineered with a CD4-based CAR produced long-lived CAR progeny that persisted for years and suppressed HIV in non-human primates (NHPs) far more effectively than conventional CAR-T infusions [386]. Likewise, CBLB-KO in HSCs enhanced cytotoxic effects of NK-progeny [387]. Other strategies use HSC editing to insert protective alleles or delete specific surface markers, enabling resistance to targeted therapies that would otherwise harm healthy cells. For example, CCR5-KO in HSCs generates HIV-resistant progeny, offering a potential autologous cure strategy [29,388]. In parallel, base editing of the CD45 epitope preserves its signaling while preventing recognition by CD45-CARs, making pan-leukocyte CAR therapy compatible with an edited hematopoietic graft [389]. A comparable approach, now in first-in-human trials for acute myeloid leukemia (AML), involves CD33 KO in donor HPCs to generate a myeloid progeny resistant to CD33-targeted cytotoxic therapies [390].

Traditionally, HSC editing strongly relies on EP for CRISPR delivery. However, Vavassori et al. demonstrated that EP alone significantly upregulated p53 target genes, such as *CDKN1A* (p21), with cumulative effect when combined with nuclease-induced DSBs and AAV6-delivered HDR templates, leading to impaired viability, growth, and clonogenic potential [116]. In contrast, GenVoy-LNPs elicited markedly lower p53 activation limited solely to editing-induced DSBs, rather than the LNP transfection process itself. Moreover, LNPs achieved editing efficiencies comparable to or exceeding those of electroporation in key progenitor subsets (i.e., CD34^+^CD133^+^CD90^+^), reaching ∼50% B2M KO while preserving cell viability, growth kinetics, and colony-forming capacity. These findings highlight that LNPs are more compatible with HSPC biology, offering efficient gene editing with reduced stress and preserved stem cell functionality. In another study, LNPs have been employed to induce KOs in CD45 and CD33 at rates of 80% and 90%, respectively, while preserving >95% viability and strong proliferative capacity relative to untreated controls [337].

True advantage of LNPs lies in their capacity for direct *in vivo* applications, bypassing the complexity and cost associated with *ex vivo* manipulation and transplantation of HSCs, as exemplified by Zynteglo gene therapy for β-thalassaemia, which was withdrawn from European markets due to its cost of US $1.8 million per patient [391]. One way to access the HSC niche in the bone marrow (BM) is through *in situ* delivery via intrafemoral (i.f.) injection of LNPs, as shown by Banda et al., who achieved local transfection, with up to ∼40% tdTomato + expression in LT-HSCs, with comparable transfection rates across non-hematopoietic niche populations such as endothelial and mesenchymal stromal cells [392]. Extending this, study by Xu et al. reported the development of antibody-free, systemically administered LNPs encapsulating ABE8e mRNA and sgRNA targeting the HBG promoter, enabling potent base editing of HSPCs within the bone marrow (BM) niche of engrafted mice [393]. By optimizing ionizable lipid chemistry (e.g., Lipid-168) and incorporating miR-122T into the mRNA cargo, they enhanced BM specificity while minimizing liver editing (from 70% to 19% with miR-122T in 3′-UTR). This strategy achieved up to 43% base editing in β-thalassemia HSCs, with robust γ-globin induction in erythroid progeny and sustained presence of the edited allele across multilineage hematopoietic outputs, even after secondary transplantation. Likewise, Siegwart's group generated BM-homing LNPs by adding covalent lipids and crosslinkers, enhancing ApoE adsorption, a key determinant of BM uptake. Their LNPs, enabled ∼5.2% CRISPR and ∼2.4%, base editing in HSCs in a sickle cell disease model, and ∼18% Cre-mediated recombination in leukemic stem cells in an aggressive MLL-AF9 leukemia model [394]. Another non-targeted approach relying on formulation screening and optimization was described by Dahlman's group, where they identified LNP67 (containing the cationic helper lipid DOTAP) as capable of passively targeting CD34^+^ HSCs in mice and rhesus monkeys, achieving up to 50% mRNA delivery in murine LT-HSCs and strong luciferase expression in CD34^+^ bone marrow cells at low doses in rhesus monkeys [395]. Nonetheless, despite preferential BM delivery with these approaches, substantial transfection in liver and spleen was still observed, indicating room for improved tissue selectivity. To improve the specificity, Shi et al. developed CD117-targeted LNPs, achieving ∼90% *in vivo* editing of HSPCs and LT-HSCs (vs. ∼25% with untargeted LNPs) and robust multilineage progeny editing (90% myeloid, 70% B cells, 50% T cells, ∼100% erythrocytes). Despite efficient BM targeting, there was still notable accumulation observed in the liver and lungs, highlighting the need for further biodistribution refinement or de-targeting approaches. Using the same CD117-targeted LNP strategy, Breda et al. achieved ∼55% tdTomato expression in LT-HSCs following Cre mRNA delivery, outperforming untargeted controls (∼19%) [396]. Unlike the previous approach, they incorporated miR-122 target sites into the mRNA, lowering hepatic editing from ∼75% to ∼20%. They further demonstrated that delivering pro-apoptotic PUMA mRNA via CD117-LNPs enabled selective depletion of HSCs and non-genotoxic conditioning, facilitating successful donor cell engraftment without relying on conventional and invasive methods such as chemotherapy or irradiation. In a conceptually distinct approach, Palanki et al. leveraged the transient liver residency of fetal HSCs and developed DoE-optimized, CD45-targeted C14-490 LNPs for *in utero* delivery of CRISPR-Cas9 mRNA [397]. They achieved ∼8% gene editing with CRISPR and ∼30% Cre-mediated recombination in fetal HSCs using targeted LNPs, compared to ∼3% with untargeted controls. While this modular platform allows substitution of cargo for other disease targets, clinical translation is limited by the invasiveness of fetal delivery and human-specific developmental constraints.

Similarly to T cells, Tessera Therapeutics has developed CD117-targeted LNPs for systemic HSC editing. In humanized mice and NHPs via i.v. administration, they achieved ∼95% GFP mRNA delivery and 62% installation of the HBB Makassar variant (correcting sickle cell disease), in humanized mice and 24% in NHPs, both with long-term, multilineage engraftment and preserved stem cell function. Repeated dosing linearly increased editing, and they also achieved ∼75% B2M KO in NHP, showcasing the platform's modularity [63,340].

*In vivo* HSC gene editing via LNPs holds major promise for immunotherapy applications, such as generating HIV-resistant cells or engineered immune effectors. However, reprogramming of long-lived, multipotent stem cells with gene editing technology may pose safety and specificity challenges, demanding precise delivery, restricted expression, and robust safety switches. Despite these hurdles, successful applications in monogenic disorders highlight the feasibility of this approach which will likely catalyze further innovation, pushing the boundaries of delivery technologies, safety engineering, and clinical translation.

### 5.5 Macrophages

Macrophages are critical actors in the innate immune system and bridge innate with adaptive immunity. They perform essential functions such as phagocytosis, cytotoxicity, antigen presentation to T cells, and orchestration of inflammation responses through cytokine secretion. Circulating monocytes infiltrate damaged or infected tissues and differentiate into macrophages or dendritic cells, while tissue-resident macrophages (e.g., Kupffer cells in the liver, microglia in the brain, osteoclasts in bones) preserve homeostasis and orchestrate repair. Phenotypically, macrophages are extraordinarily plastic, and traditionally, they are classified into two major subsets: activated, pro-inflammatory M1, and cytotoxic, anti-inflammatory, pro-repair M2. Due to this plasticity and wide range of functions, macrophages are implicated in various diseases, including cancer, autoimmunity, neurodegeneration, and cardiovascular disorders. In cancers, tumor-associated macrophages (TAMs) often dominate the immune infiltrate. In early stages, they exert cytotoxic M1-like activity, but progressively become immunosuppressive, pro-angiogenic M2-like TAMs. This plasticity offers a therapeutic opportunity: in cancer, M2 TAMs can be reprogrammed to an anti-tumoral M1 phenotype, while in autoimmune or chronic inflammatory settings, promoting an M2 phenotype helps mitigate damaging inflammation [398].

Building on this versatility, therapeutic approaches involve usage of CAR macrophages (CAR-Ms), which harness the cells' natural abilities, such as phagocytosis, matrix degradation, and antigen presentation, toward defined tumor targets. Pre-clinical data and early clinical trials show that CAR-Ms infiltrate solid tumors, engulf antigen-positive cells, repolarize by-stander TAMs to an M1 phenotype, recruit cytotoxic lymphocytes and do so with lower cytokine-release toxicity than CAR-T strategies [399,400]. In parallel, other common therapeutic approaches that can be in synergy with CAR-M or checkpoint inhibitors, seek to improve their anti-tumor function by either reprogramming M2 TAMs into M1 phenotype or by disruption of “don't-eat-me” CD47-SIRPα signaling with siRNA, short hairpin RNA (shRNA), antibodies or small molecule inhibitors to enhance phagocytosis of CD47^+^ tumor cells [[401], [402], [403]].

CRISPR-based gene editing permits more permanent phenotypic reprogramming. Strategies include: (i) integrating a CAR construct into a safe locus; (ii) knocking out SIRPα to enhance phagocytosis of CD47^+^ tumor cells; and (iii) promoting fixed M1 or M2 phenotypes depending on therapeutic needs. Given macrophage plasticity, genetically locking cells into a desired state is increasingly important. For example, KO of *Prkacb* or *Pik3cg* promotes a permanent M1 state [404]. This was demonstrated in a recent study employing lipidated, protoplast-derived nanovesicles targeted to TAMs to deliver Cas9 RNPs against *Pik3cg*, along with TLR9-stimulating CpG DNA, achieving 34% *in vivo* gene editing efficiency and effective M1 reprogramming [405].

Traditionally, macrophage engineering strongly relies on viral vectors, especially on adenoviral vectors (Adv) as they intrinsically favor the M1 phenotype [402,404]. However, their immunogenicity greatly hampers *in vivo* applications. Recent innovations include epigenetic reprogramming using CRISPR/dCas9-EZH2 delivered via lentivirus: silencing Hif1α in TAMs led to durable M1 reprogramming after cell transfer *in vivo*, with inhibition of tumor growth, enhanced T cell activation, and reduced angiogenesis [406]. Using optimized nucleofection of Cas9-RNP complexes, Freund et al. achieved >90% KO efficiency in primary murine and human macrophages, including simultaneous disruption of up to three genes [407]. However, this approach leads to variable viability post-EP (∼20% to >80%) and required extensive optimization.

To avoid viral vectors, lipid- and polymer-based nanoparticles have emerged as viable tools for non-viral mRNA delivery to macrophages. In this context, the study by Moradian et al. systematically evaluated lipid- and polymer-based mRNA delivery systems in primary macrophages *ex vivo*, identifying lipid-one as superior in balancing transfection efficiency and immune activation, especially when combined with modified nucleotides [408]. Building on this, SM-102-based LNPs achieved efficient *ex vivo* and *in vivo* RNA delivery in primary macrophages, including >90% transfection and potent gene knockdown, all while enabling antibody-guided targeting of specific macrophage subsets [409]. Similarly, phosphatidylserine-modified SM-102 LNPs transfected nearly 100% of macrophages *in vitro* and, upon i.v. injection, significantly increased macrophage transfection *in vivo* (∼19% vs. ∼7% with unmodified LNPs), although off-target uptake in the spleen and neurons remained, and no selectivity between M1 and M2 subtypes was observed [410]. Moreover, van der Meel's team pioneered apolipoprotein A1-based nanoparticles (aNPs) that integrate various cargos into LNP-like architecture functionalized with apoA1, mimicking natural lipoprotein trafficking to enable systemic delivery to myeloid cells and bone marrow progenitors *in vivo* [186]. In a distinct approach, Tang et al. co-delivered CAR mRNA and siRNA via LNPs decorated with CRV peptide, achieving *in situ* macrophage reprogramming to eliminate MRSA in sepsis model [411]. In contrast to antibody- or peptide-guided systems, Yang et al. introduced a corona-assisted delivery strategy using PPZ-A10 LNPs to reprogram liver macrophages with CAR and Siglec-GΔITIMs mRNAs. This approach achieved preferential, though not exclusive, macrophage targeting *in vivo* and led to anti-tumor effects in an aggressive hepatocellular carcinoma model [412]. The potential of this *in vivo* approach is highlighted by joint work of Carisma Therapeutics and Moderna, as well as Myeloid therapeutics in partnership with Acuitas, who are developing LNP-based *in vivo* CAR-M therapies for multiple solid tumors and autoimmune diseases with first results in patients (e.g. NCT06478693).

Recent efforts extend beyond mRNA delivery to include gene and RNA editing using nanoparticles. PBAE29-based nanoparticles (based on branched poly(β-amino ester)), functionalized with carboxylated mannan (targeting CD206) and using macrophage-specific promoters (SP146), were used to deliver minicircle plasmids encoding Cas9 and CasRx (RNA-targeting nuclease) [413]. These enabled dual RNA knockdown of SIRPα and Siglec-10, two key phagocytosis checkpoint receptors, enhancing macrophage phagocytosis and antigen presentation, leading to greater CD8^+^ T cell activation and reduced tumor burden in multiple mouse models. *In vitro* transfection efficiency for CasRx minicircle plasmids reached ∼30%, and *in vivo* i.p. delivery increased the proportion of double-negative (SIRPα^−^Siglec-10^-^) TAMs from ∼43% to ∼82%. In mouse models of ovarian, pancreatic and liver tumors, dual RNA editing increased macrophage phagocytosis of tumor cells and enhanced antigen presentation, resulting in stronger CD8^+^ T cell activation and reduced tumor burden. However, these results were achieved through intraperitoneal delivery, which raises translational questions regarding applicability to other tumor types and scalability to larger animal models or systemic administration in humans. Nonetheless, this study provides a compelling proof of concept for macrophage-specific RNA editing (*MAGE*) using a non-viral delivery system that integrates targeted delivery, minicircle plasmid design, a macrophage-specific promoter, and CasRx-mediated knockdown, highlighting a modular and cell-selective approach with promising therapeutic potential. Similarly, Wang et al. employed mannose-functionalized cationic nanoclusters embedded in a ROS-responsive hydrogel for the localized delivery of Cas13 RNPs, achieving efficient RNA-level knockdown of osteopontin in inflammatory macrophages and successfully reprogrammed the injury microenvironment, attenuating fibrosis and enhancing tissue repair [414].

Although LNPs have been successfully used to deliver various cargos to macrophages *in vivo*, CRISPR-based gene editing in these cells using LNPs remains unachieved. Nevertheless, given the availability of macrophage-targeting strategies and emerging tools to enhance specificity and control, adapting LNP platforms for precise and efficient gene editing in macrophages appears to be a promising next step in therapeutic development.

### 5.6 Dendritic cells

Dendritic cells (DCs) are professional antigen-presenting cells that initiate and shape adaptive immune responses. They capture antigens, migrate to lymph nodes, and present processed peptides to T cells, providing the necessary co-stimulation signals to prime both CD4^+^ and CD8^+^ T cell responses [415]. This central role makes them crucial in cancer immunotherapy, infectious diseases vaccination, and the regulation of immune tolerance or in breaking tolerance in autoimmunity. DCs have been used as vaccines: patient-derived DCs loaded with tumor antigens can elicit anti-cancer T cell responses [416]. This was utilized in various scenarios including TriMix-DC vaccination, which primes antigen-specific T cells in melanoma, as well as FDA-approved sipuleucel-T for prostate cancer [417,418]. However, the effectiveness of DC vaccines is often constrained by their functional state. The upregulation of inhibitory molecules (e.g., PD-L1) or insufficient co-stimulatory signaling can result in suboptimal T cell priming [419]. Therefore, DC can be genetically engineered to remove immunosuppressive pathways (e.g., PD-L1, IDO); express stimulatory cytokines or modify the antigen-processing and presentation machinery [420]. In contrast, for autoimmune diseases, transplant rejections and inflammation, DCs can be engineered to enhance their tolerogenic properties, promoting regulatory T cell (Treg) induction and immune tolerance [421].

Several studies have already demonstrated the feasibility of CRISPR-based gene editing in DCs. For instance, EP of Cas9-RNPs has achieved KO efficiencies of approximately 90% in both murine and primary human monocyte-derived DCs. However, this approach required careful protocol optimization and was associated with reduced viability in certain subsets, such as CD24^+^ DCs, where viability dropped by around 30% [407]. Similarly, Cas9-RNP nucleofection was used to target *Ndrg2*, a gene known to constrain regenerative capacity in murine DCs, achieving up to 90% editing efficiency. When these edited DCs were delivered in hydrogel scaffolds to murine models, they significantly accelerated wound healing [422].

DCs are relatively easily transfected with lipid-based nanoparticles, with numerous demonstrations of successful applications both *ex vivo* and *in vivo*. Among several methods tested in primary human DCs, lipid-based transfection proved most effective for gene silencing, with preserved viability and minimal activation, outperforming electroporation, viral vectors, and other chemical reagents [423]. *In vivo* targeting of DCs with LNPs has been successfully demonstrated by targeting mannose receptors (CD206, DC-SIGN) or specific lectin receptors (DEC-205 and Clec9a) using either mannose-functionalized LNPs (and lipopolyplexes) or antibody-mediated strategies [177,424]. More recent study employed LNPs with ginsenoside Rg3 with dual role as cholesterol analog and targeting system for GLUT1 receptors on DCs thereby enhancing DC transfection efficiency *in vivo* [425].

While lipid-based systems dominate mRNA delivery, most DC gene editing studies have used polymeric systems. For instance, PEG-b-PLGA-based cationic lipid nanoparticles (CLANs) were used to deliver Cas9 mRNA and CD40-targeting gRNA into DCs both *in vitro* and *in vivo*. Despite moderate editing (∼25% *in vitro*, ∼11% *in vivo*), CD40 disruption lowered costimulatory signals, reduced T cell activation, and slightly extended graft survival [426]. Wan et al. developed a microneedle-based system for the co-delivery of polymer-encapsulated Cas9 RNPs and dexamethasone PLGA nanoparticles to target the *NLRP3* gene in dendritic cells and skin-resident immune cells. This approach achieved up to 36% editing (DC2.4 cells) *in vitro* and 21% *in vivo*, improving inflammatory skin outcomes by mitigating glucocorticoid resistance. Therefore, it offers a distinctive, localized and synergistic strategy for immune modulation via genome editing [180].

While LNPs are widely used to transfect DCs with mRNA, LNP-mediated CRISPR delivery remains underexplored. However, recent advances show promise. Mao et al. optimized distinct class of LNPs termed BLANs (BAMEA-O16B lipid-assisted nanoparticles) by modulating cholesterol content which significantly enhanced mRNA transfection and gene editing in DCs (e.g., ∼28% vs ∼63% RFP^+^ cells and ∼16.6% PD-L1 knockout *in vitro*). Intratumoral administration of low-cholesterol BLANs led to substantial reduction of PD-L1 expression in DCs, promoted T cell activation, and suppressed tumor growth. However, editing efficiency *in vivo* was not rigorously quantified, and off-target effects in DC subsets remain unclear [179]. While LNP-based strategies for dendritic cell editing remain limited, they are beginning to expand the experimental toolkit for studying and modulating immune responses.

Overview of CRISPR-Cas–mediated genome editing strategies applied to key immune populations, including T cells, NK cells, B cells, dendritic cells, macrophages, and HSCs. Editing strategies encompass loss-of-function (e.g., gene knockouts to disrupt inhibitory pathways), gain-of-function (e.g., insertion of CARs or cytokine genes), and cell-type–specific applications such as allogeneic T cell generation, antibody reprogramming in B cells, and functional remodeling of macrophages. Multipotent HSCs serve as a versatile platform for generating engineered lineage-specific immune subsets.

## 6 Leveraging artificial intelligence for precision delivery and optimization of CRISPR-LNP systems

The next generation of LNPs for CRISPR delivery is being shaped by the combined power of high-throughput compound generation, multi-omics, and artificial intelligence (AI). With thousands of possible lipid structures and complex LNP-cell interactions, machine learning (ML) is pivotal in deciphering structure–function relationships and accelerating rational design. These new approaches link high-throughput synthesis, virtual lipid libraries, and ML models to reveal structure-function relationships and predict how nanoparticles behave in transfections. These tools enable a shift from traditional trial-and-error toward data-driven “fit-for-purpose” LNP design that integrates AI to decode cell-specific delivery patterns, optimize formulation and production parameters, and accelerate the automated discovery of novel lipid components [427].

To elucidate how LNP structure dictates cell-specific delivery, Dahlman's group developed a high-throughput DNA barcoding system that enables simultaneous testing of hundreds of LNP formulations *in vivo*. This approach generated large-scale datasets capturing how subtle chemical modifications influence delivery profiles, ultimately enabling the identification of structure–function relationships that govern tissue- and cell-type-specific tropism [143,428]. AI models trained on these datasets can uncover structure–activity relationships, advancing rational LNP design. Complementing this, Boehnke et al. used multiomic screening and ML to systematically map NP-cell interactions across 488 cancer cell lines, identifying biological determinants such as gene *SLC46A3* expression that predict LNP uptake and transfection *in vitro* and *in* *vivo* [429]. This strategy enables predictive modeling of cell-specific delivery based on molecular markers.

Beyond cellular uptake, ML has also been employed to predict the composition of the protein corona, the layer of proteins adsorbed onto nanoparticles upon exposure to biological fluids. ML models trained on proteomic data and nanoparticle physicochemical properties have helped predicting protein corona composition and its downstream impact on cellular recognition, a key factor in engineering LNPs with consistent bio–nano interfaces [430].

AI also accelerates formulation and manufacturing optimization through integration with Design of Experiments (DoE) frameworks. For example, microfluidic manufacturing of liposomes has benefited from AI-guided DoE, which efficiently tunes parameters such as lipid composition and flow rates to improve scalability and reproducibility [431]. Hanafy et al. applied this approach to optimize lipid ratios, predicting transfection outcomes (R^2^ > 0.8) across immune cell types including Jurkat T cells, THP-1 monocytes, and dendritic cells, while minimizing hepatocyte expression [432]. Notably, reducing cholesterol content (5–20%) and increasing phospholipid proportion (32–50%) significantly suppressed off-target liver expression while maintaining efficacy in immune cells. *In vivo*, these formulations showed significantly reduced liver signal and preferential spleen expression. Despite being promising, the resolution was limited by bulk tissue imaging and cell line models.

Anderson's group applied deep learning models trained on large *in vitro* and *in vivo* datasets to screen millions of *in silico*-designed ionizable lipids, identifying candidates with improved mRNA delivery to immune cells and challenging tissues [433,434]. Several ML-prioritized lipids, when synthesized, such as 119-23, FO-32, and FO-35, outperformed clinical benchmarks (SM102-and MC3-based LNPs) in large-animal models, including ferrets.

Li's group introduced the AGILE platform, combining pre-trained models, DoE, and wet-lab validation to rapidly identify ionizable lipids with cell-specific tropism. The AGILE-derived formulation R6 exhibited superior delivery to macrophages over MC3-based controls [435].

Pushing the frontier further, Li's team also introduced the LUMI-lab system – an autonomous, closed-loop AI platform integrating deep learning, robotic synthesis, and high-throughput screening [436]. This platform marks a shift toward autonomous therapeutic discovery by integrating pretrained molecular models, robotic experimentation, and active learning into a fully automated pipeline for ionizable lipid synthesis, LNP formulation, and *in vitro* screening. Over ten iterative cycles, LUMI-lab synthesized and tested more than 1700 ionizable lipids, autonomously uncovering brominated tail motifs that enhanced mRNA delivery. Its top-performing lipid, LUMI-6, enabled more than 20% CRISPR-Cas9 gene editing in lung epithelial cells following inhalation which is the highest efficiency reported for inhaled LNP delivery. It demonstrated how AI can extract new design principles in underexplored chemical spaces with little prior data. Despite its scale (∼221k lipid structures), the library remains constrained to Ugi-component chemistry, limiting broader chemical diversity. And even though current outputs rely on simple transfection readouts without functional, immune assays, the entire framework offers immense potential. Future extensions could adapt LUMI-lab to immune cell targeting by integrating primary cell models, organoids, or more complex readouts such as endosomal escape and immune activation. As such, this platform sets the stage for the next generation of AI-guided LNP engineering.

In parallel, AI is advancing genome editing technologies themselves. Tools like DeepCRISPR, CRISTA, and CRISPRon incorporate sequence features, chromatin context, and Cas variants to improve guide RNA specificity and reduce off-target effects [437]. SPROUT, for instance, predicts repair outcomes in primary T cells [438]. AI is also advancing the design of high-fidelity genome editors, such as prime editing, with tools like Easy-Prime and PrimeDesign enabling pegRNA design and efficiency prediction. Notably, PRIDICT2.0 (trained on over 400,000 pegRNAs) and its chromatin-aware tool ePRIDICT use deep learning to predict editing efficiency across diverse edits and loci, integrating both sequence and epigenetic context [439]. AI is also being applied to protein engineering – for instance, using AlphaFold2 to design Cas variants with enhanced fidelity and specificity [437]. Beyond individual tools, CRISPR-GPT represents a shift toward fully integrated AI copilots that orchestrate end-to-end gene-editing workflows [440]. Built on a multi-agent large language model (LLM) architecture, it enables experimental planning, gRNA design, delivery method selection, protocol drafting, and data analysis through interactive reasoning and tool integration. Notably, the system uses expert-curated data and a retrieval-based approach to suggest delivery methods suited to each cell type and biological context, including difficult-to-transfect cells. While CRISPR-GPT demonstrates how LLM-driven systems can potentially unify fragmented workflows and address both computational and experimental bottlenecks in genome editing, a key limitation must be acknowledged: current validations are restricted to well-characterized human cell lines, and the system has not yet been tested in primary immune cells, which is the population most relevant to the therapeutic applications discussed in this review. Extending CRISPR-GPT to primary T cells, NK cells, and HSCs, as well as to *in vivo* delivery contexts, therefore represents a critical and immediate future challenge for the field.

**Fig. 8:**
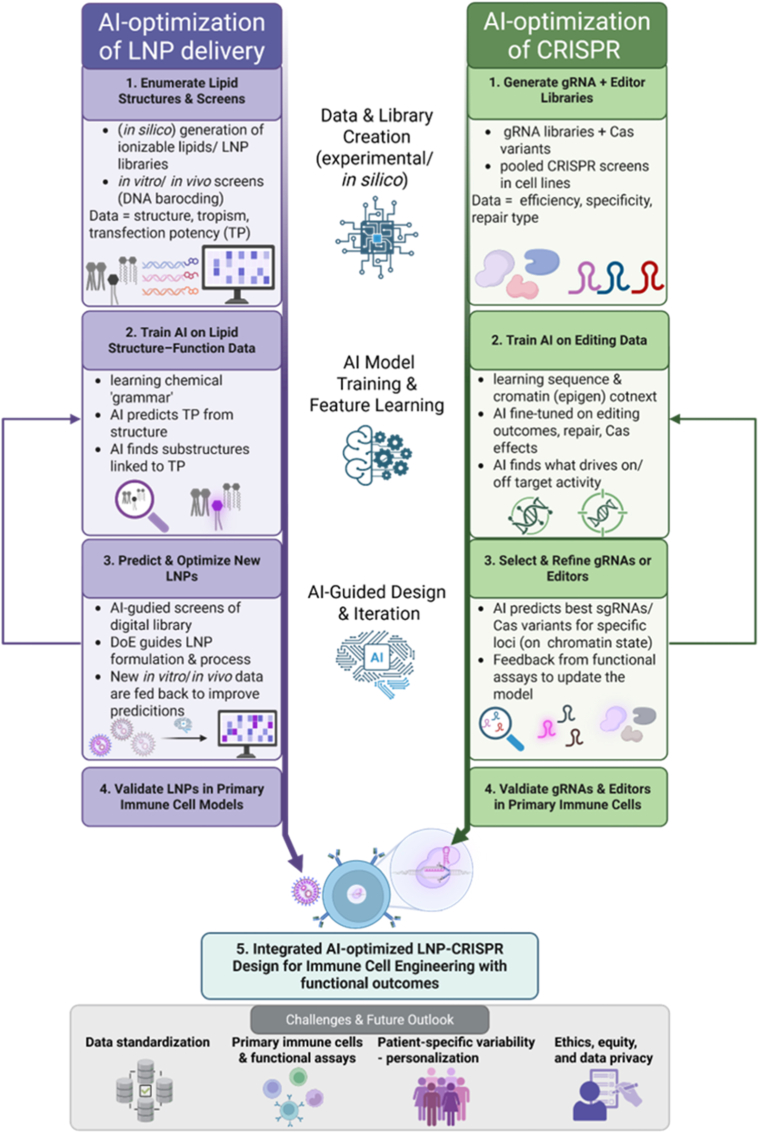
AI-driven design of LNP–CRISPR platforms for immune cell engineering.

AI has undoubtedly huge potential for accelerating the design of both LNP-based delivery and genome editors, with significant milestones already achieved in preclinical research (Fig. 8). Yet, the translation of these AI-guided platforms into clinically viable immunotherapies remains constrained by several critical hurdles. AI models are only as good as the data they are trained on and, currently, most datasets lack standardization, high-resolution metrics, and relevance to primary immune cells. Translatability is further limited by a reliance on immortalized cell lines and simple readouts (e.g., transfection efficiency), which do not capture the complexity of immune activation, proliferation, or function, especially after *in vivo* translation. Beyond predicting CRISPR delivery success, models must account for patient-specific cellular variability and downstream behavior, for instance how edited immune cells like NK or T cells function and persist *in vivo*. Looking forward, AI could help patient stratification and personalize dosing or delivery strategies in trials. However, such advances rely on access to sensitive omics data, raising concerns about privacy, data ownership and equity.

Schematic workflow of parallel AI-driven pipelines for optimizing LNP delivery systems (left) and CRISPR-based gene editing tools (right). Each pipeline involves the generation of structural or functional variant libraries, model training using experimental and computational (*in silico*) datasets, and iterative design–test–refine cycles. Integration of both pipelines supports the rational co-optimization of LNP–CRISPR systems tailored for precise and efficient immune cell engineering. Key future challenges include data standardization, modeling in primary immune cells, addressing inter-patient variability, and navigating ethical and regulatory considerations.

## 7 Discussion, perspectives and conclusion: trends, clinical translation and future directions

Gene editing is already reshaping modern medicine, enabling the correction of genetic disorders, the reprogramming of immune cells, and more, with even greater potential still ahead. But clinical success depends not only on the molecular precision of gene editors, but also on their ability to reach their targets. Yet the translation of this promise into clinical reality rests on a pivotal and persistent challenge: delivery. Precision at the molecular level means little without precision at the systemic level. Gene editors must not only be effective, but also delivered to the right cells, at the right time, with clinical-grade consistency and scalability.

LNP-based CRISPR delivery is emerging as practical response to this need. Building on the momentum of mRNA COVID-19 vaccines, LNPs are now being adapted to deliver genome editors across diverse applications: in immunotherapy, metabolic, genetic, and ophthalmologic disorders. Even though this landscape is dominated by viral vectors, we are witnessing a shift due to safety concerns, manufacturing limitations, and increased interest toward non-viral alternatives. This shift is exemplified by the work of Hołubowicz et al., who achieved precise genome correction in retinal disease using LNP-delivered base and prime editor RNPs. Building on their earlier reliance on viral vectors, the authors highlight how LNPs enable a more defined and clinically relevant formulation through direct RNP delivery [126]. And while AAVs remain foundational to many approved therapies, declining investment, recurring clinical shortcomings and strategic pullbacks have dampened enthusiasm. In contrast, LNPs are gaining traction as evidenced by projected market size of LNP-enabled genetic medicines of around $48 billion by 2036 (by the detailed analysis as of December 2021) [441]. This transition is reflected in the momentum across the biotech landscape, where LNP-CRISPR platforms are advancing rapidly, from clinical-stage efforts by Intellia, Verve (acquired by Eli Lilly for $1.3B in June 2025) and CRISPR Therapeutics to preclinical innovation at companies like Beam, Prime Medicine, and ReCode. A powerful testament to this clinical potential is the recent case in which LNPs enabled effective *in vivo* base editing in a pediatric patient, resulting in a life-saving outcome [127].

With both preclinical milestones and early clinical trials now targeting immune cells, LNP-based CRISPR delivery is being positioned as a leading contender in the push to translate immune cell editing into effective therapies. Some of the most instructive lessons so far in genome editing and immune cell-specific delivery have come from HSCs and T cells. In HSCs, gene editing is being explored as a curative strategy for rare monogenic disorders, which is an area where there are often no viable alternatives, and regulatory pathways can be more streamlined. In T cells, the success of CAR T cell therapies has established both clinical and commercial validation, catalyzing rapid progress around them [442]. Together, these two cell types have driven innovation in both editing technologies and delivery systems. Capstan Therapeutics, acquired by Abbvie for $2.1B in June 2025, exemplifies this. Its first-in-human trial employs antibody-targeted LNPs to generate CAR T cells *in vivo* to replace costly and complex *ex vivo* manipulation. This marks a paradigm shift, one that may soon extend, not only to other immune populations, but also towards immune cell gene editing *in vivo*. Looking forward, immune populations such as NK cells, B cells, and myeloid cells represent the next frontier. Their potential spans oncology, autoimmune disease, and even vaccine enhancement, but their heterogeneity makes precision delivery even more critical. Here, LNPs, especially when engineered with modular, antibody-guided, or AI-optimized designs, could play a defining role.

Meanwhile, industry continues to shape the pace and direction of innovation. According to Cowen's 2023 industry overview, more than 90 clinical-stage gene editing programs are currently underway, with immune cell applications among the fastest-growing sectors [443]. Industry analysts broadly project that the global cell and gene therapy market will continue to expand rapidly, with estimates surpassing $100 billion by the early 2030s. Dozens of companies, including CRISPR Therapeutics, Caribou, Editas, Beam, Nkarta, ONK Therapeutics, and others, are translating LNP-based delivery platforms into trial-ready therapies.

Yet despite growing promise, translation to the clinic remains uncertain and it poses a relevant question: “Can we avoid repeating the pattern where promising preclinical breakthroughs consistently fail to translate into human success?” This concern is especially present in immune cells, where editing efficiency is shaped not only by payload and delivery, but also by contextual factors, such as activation state, differentiation stage, and the inherent variability of donor-derived samples. Another underlying issue is the tendency toward a one-size-fits-all delivery approach. Given the diversity of immune cell types and therapeutic goals, this is unlikely to succeed. Instead, progress will depend on rational, fit-for-purpose LNP design, tailored to cell subtype, route of administration, and desired outcome.

AI can accelerate this shift by enabling predictive modeling of LNP structure, biodistribution, and editing outcomes, speeding up the process and potentially reducing the cost. But delivery tools are only as reliable as the systems we test them in. As George Box stated, “All models are wrong, but some are useful” [444]. In immune engineering, however, some are particularly inadequate. The translational gap is often rooted in model mismatch: for results to be meaningful, both *ex vivo* and *in vivo* models must align with clinical realities. In immune cell engineering, this means using activation and transfection protocols that must be not only efficient but also translatable to clinical-grade manufacturing and patient-derived material. Too often, preclinical models are designed to show maximized (editing) efficiency rather than mirror therapeutic feasibility, leading to false optimism. Target immune cells in patients – particularly in cancer, autoimmunity, or post-chemotherapy settings – are often stressed, dysfunctional, or altered by prior treatments. Whether such cells can be edited with comparable efficiency or retain normal function post-transfection remains a critical question, often overlooked in preclinical design. To bridge this translational gap, the field must invest in developing more predictive and scalable immune-relevant models that more accurately reflect the biological complexity and therapeutic realities of human immune systems, beyond current organ-on-chip tools and humanized mice. Platforms like MIMIC®, which use fully autologous human immune cells, provide a valuable bridge between *in vitro* discovery and *in vivo* application, capturing the complexity of donor-specific variability and immune responses [445]. The use of primary human cells enables modeling of *in vivo* phenomena such as immunosenescence and donor-specific variability in antibody and T cell responses, both of which critically influence therapeutic efficacy. By capturing this biological diversity, such models provide a powerful platform for evaluating delivery and editing strategies in clinically relevant settings.

The field is advancing rapidly, driven by major innovations in gene editing, delivery technologies, and immune cell biology. Yet innovation alone is insufficient. Meaningful progress will depend on translating immune cell editing into therapies that are safe, scalable, and clinically reliable.

An equally important consideration is the development of robust Chemistry, Manufacturing, and Controls (CMC) frameworks. The global deployment of mRNA-LNP vaccines during the COVID-19 pandemic demonstrated that LNPs can be manufactured reproducibly at industrial scale with acceptable batch-to-batch consistency, stability, and clinical performance. Advances in microfluidic formulation technologies have further improved control over particle size distribution, encapsulation efficiency, and formulation reproducibility, helping establish scalable GMP-compatible manufacturing pipelines for nucleic acid therapeutics.

Extending these standards to LNP-CRISPR systems remains more challenging. Compared with conventional mRNA-LNP formulations, CRISPR payloads often involve larger or multi-component cargos, including Cas mRNA with sgRNA or RNP complexes, each with distinct physicochemical and stability requirements.

Maintaining consistent payload integrity, editing activity, and storage stability during manufacturing and scale-up therefore becomes substantially more demanding. In addition, increasingly sophisticated formulations incorporating targeting antibodies, peptides, or novel ionizable lipids may improve delivery specificity, but they also introduce additional complexity in characterization, comparability testing, and regulatory evaluation.

Therefore, innovation must remain grounded in application with real-world patients in mind. Strategies that are too disruptive in embracing complexity, though theoretically powerful and innovative, still tend to fail under the weight of real-world constraints of scale-up, reproducibility, and regulatory burden. In contrast, simpler systems based on validated components tend to navigate the translational path more effectively. This principle is illustrated by Capstan Therapeutics, which is developing targeted mRNA-LNP approaches that combine established therapeutic modalities, including antibodies and LNP-based mRNA delivery, to support a more defined development pathway. A complementary challenge is addressed by the NANOSPRESSO initiative, which focuses on decentralized manufacturing of RNA-LNP gene-editing therapies. By enabling on-demand production in hospital settings, such approaches aim to reduce logistical barriers and better align advanced RNA-based therapeutics with point-of-care clinical implementation. Time will tell whether these approaches are truly sustainable over the long term without major obstacles emerging at different stages of the manufacturing and the clinical trials.

Yet the long-term vision is compelling. The combination of AI-guided design, next-generation genome editors, and deeper understanding of immune cell biology is set to transform cell engineering. If tailoring delivery systems like LNPs to T cells, NK cells, HSCs, or myeloid subsets can continue evolving in tandem with these technologies, they may well become the foundation of a new era in cell and gene medicine, one grounded in real, patient-centered progress.

In the near term, *ex vivo* LNP-mediated gene editing of immune cells is likely to progress incrementally toward clinical implementation, particularly in settings where transient, non-viral delivery can address current manufacturing or safety limitations. The path toward *in vivo* immune cell editing is less immediate and will probably depend, in part, on continued clinical validation of LNP-based therapies in the liver, where this delivery modality is currently most advanced. Extension beyond hepatic targets will require substantial improvements in cell-selective delivery, tissue distribution, endosomal escape, and evasion of immune clearance. These challenges are considerable, and their resolution is likely to occur over years rather than months. Nevertheless, LNPs remain a plausible foundation for programmable *in vivo* immune cell therapies, if formulation design, payload selection, dosing strategy, and safety monitoring are systematically optimized for the relevant immune cell subset and disease context.

In conclusion, immune cell editing has moved from a primarily experimental field toward a clinically actionable therapeutic strategy. Its broader implementation will depend on the development of LNP platforms that combine efficient delivery, predictable biodistribution, manufacturability, and an acceptable safety profile. When these requirements are met, LNP-enabled immune cell engineering could contribute to a new generation of targeted and adaptable cellular therapies.

## CRediT authorship contribution statement

**Ivan Ciganek:** Conceptualization, Data curation, Investigation, Methodology, Writing – original draft. **Christophe Delehedde:** Data curation, Investigation, Writing – original draft. **Anne Galy:** Funding acquisition, Validation, Writing – review & editing. **Michel Cogné:** Formal analysis, Validation, Writing – review & editing. **Nathalie Rameix:** Conceptualization, Data curation, Formal analysis, Writing – original draft. **Catalina Bordeianu:** Funding acquisition, Project administration, Supervision, Writing – review & editing. **Chantal Pichon:** Conceptualization, Funding acquisition, Project administration, Supervision, Validation, Writing – review & editing.

## Declaration of competing interest

The authors declare the following financial interests/personal relationships which may be considered as potential competing interests: Chantal PICHON reports financial support and equipment, drugs, or supplies were provided by Sanofi. Anne Galy reports financial support, article publishing charges, equipment, drugs, or supplies, and travel were provided by French National Research Agency. Michel Cogne reports financial support, article publishing charges, equipment, drugs, or supplies, and travel were provided by French National Research Agency. Catalina Bordeianu reports a relationship with Sanofi that includes: employment and equity or stocks. Nathalie Rameix reports a relationship with Sanofi that includes: employment and equity or stocks. Ivan Ciganek reports a relationship with Sanofi that includes: employment. Christophe Delehedde reports a relationship with Sanofi that includes: employment and equity or stocks. If there are other authors, they declare that they have no known competing financial interests or personal relationships that could have appeared to influence the work reported in this paper.

## Data Availability

No data was used for the research described in the article.
